# Blockade of TRPC Channels Limits Cholinergic-Driven Hyperexcitability and Seizure Susceptibility After Traumatic Brain Injury

**DOI:** 10.3389/fnins.2021.681144

**Published:** 2021-08-19

**Authors:** Chase M. Carver, Haley R. DeWitt, Aiola P. Stoja, Mark S. Shapiro

**Affiliations:** Department of Cellular and Integrative Physiology, University of Texas Health San Antonio, San Antonio, TX, United States

**Keywords:** ion channels, TRPC channels, hippocampus, epilepsy, seizure, traumatic brain injury, epileptogenesis, hyperexcitability

## Abstract

We investigated the contribution of excitatory transient receptor potential canonical (TRPC) cation channels to posttraumatic hyperexcitability in the brain 7 days following controlled cortical impact model of traumatic brain injury (TBI) to the parietal cortex in male adult mice. We investigated if TRPC1/TRPC4/TRPC5 channel expression is upregulated in excitatory neurons after TBI in contribution to epileptogenic hyperexcitability in key hippocampal and cortical circuits that have substantial cholinergic innervation. This was tested by measuring TRPC1/TRPC4/TRPC5 protein and messenger RNA (mRNA) expression, assays of cholinergic function, neuronal Ca^2+^ imaging in brain slices, and seizure susceptibility after TBI. We found region-specific increases in expression of TRPC1, TRPC4, and TRPC5 subunits in the hippocampus and cortex following TBI. The dentate gyrus, CA3 region, and cortex all exhibited robust upregulation of TRPC4 mRNA and protein. TBI increased cFos activity in dentate gyrus granule cells (DGGCs) and layer 5 pyramidal neurons both at the time of TBI and 7 days post-TBI. DGGCs displayed greater magnitude and duration of acetylcholine-induced rises in intracellular Ca^2+^ in brain slices from mice subjected to TBI. The TBI mice also exhibited greater seizure susceptibility in response to pentylenetetrazol-induced kindling. Blockade of TRPC4/TRPC5 channels with M084 reduced neuronal hyperexcitation and impeded epileptogenic progression of kindling. We observed that the time-dependent upregulation of TRPC4/TRPC5-containing channels alters cholinergic responses and activity of principal neurons acting to increase proexcitatory sensitivity. The underlying mechanism includes acutely decreased acetylcholinesterase function, resulting in greater G_*q*__/__11_-coupled muscarinic receptor activation of TRPC channels. Overall, our evidence suggests that TBI-induced plasticity of TRPC channels strongly contributes to overt hyperexcitability and primes the hippocampus and cortex for seizures.

## Introduction

Traumatic brain injury (TBI) accounts for 20% of symptomatic epilepsies and 5–6% of all epilepsy (Garga and Lowenstein, 2006). TBI initiates many chronic neurological problems that persist long after injury, associated with both acute damage and compensatory brain changes during recovery. Before seizure symptoms begin, the injured brain undergoes pathophysiological changes that include excitotoxicity of neurons and increased hyperexcitability, which can contribute to seizure susceptibility (Algatta and Huang, 2013; Bramlett and Dietrich, 2015; Ng and Lee, 2019). The hippocampus is a limbic structure that heavily relies on changes in ion channel properties and expression that are key for learning and memory, and maladaptive changes after TBI are linked to cognitive dysfunction, neurodegenerative disorders, and temporal lobe epilepsy (Saatman et al., 2006; Bonislawski et al., 2007; Cohen et al., 2007; Smith et al., 2012). Even though the site of physical injury may be localized to the cerebral cortex, the hippocampus experiences selective neuronal loss and shows robust plasticity in neuronal properties within the first week after TBI, and this can lead to pathophysiological and chronic hyperexcitability (Golarai et al., 1992; Lowenstein et al., 1992; Smith et al., 1995; Witgen et al., 2005; Neuberger et al., 2017, 2019). Due to this important role in seizure susceptibility, the hippocampus serves as a critical structure in preventing posttraumatic seizures (PTS) from spreading throughout the brain.

“Canonical” transient receptor potential (TRPC) channels are non-selective cation channels that are largely activated in response to hydrolysis of phosphatidylinositol 4,5-bisphosphate (PIP_2_) by G_*q/*__11_-mediated activation of phospholipase Cβ (PLC) (Schaefer et al., 2000; Lee et al., 2003; Carver and Shapiro, 2019). The TRPC channel family is variably permeable to Ca^2+^ and Na^+^ ions, inducing both membrane depolarization and Ca^2+^-mediated second messenger cascades (Owsianik et al., 2006; Fowler et al., 2007; Storch et al., 2012; Mori et al., 2015), and channel opening is augmented by Ca^2+^/CaM as a positive-feedback mechanism (Tang et al., 2001; Singh et al., 2002; Zhu and Tang, 2004; Ordaz et al., 2005; Vinayagam et al., 2020). These channels are widely expressed in the central nervous system (CNS) neurons and have been suggested to have a role in epilepsy and cell death after seizures (Zheng, 2017). Of the TRPC subtypes, TRPC4 and TRPC5 comprise a majority of brain expression, with TRPC3, TRPC1, and TRPC6 only moderately expressed, and TRPC2 and TRPC7 demonstrating very low levels of expression (Fowler et al., 2007). TRPC1/TRPC4/TRPC5-containing heteromeric channels contribute to the modulation of neuronal excitability and synaptic transmission in the corticolimbic system, including learning and memory functions (Fowler et al., 2007; Broker-Lai et al., 2017). TRPC4/TRPC5 channel inhibition has been previously shown to convey antidepressant and anxiolytic effects, as measured by rodent behavioral tasks (Yang et al., 2015).

The function of TRPC channels in modulating epileptiform excitability has been established (Phelan et al., 2012, 2017; Zheng and Phelan, 2014; Ko and Kang, 2017), and these studies demonstrate loss of muscarinic acetylcholine receptor (mAChR)-driven excitability in TRPC knockout mice. Whereas these studies suggest the involvement of TRPC channels in epileptogenesis, the contribution of TRPC channels in acquired epilepsies and PTS has not been previously explored. It is known that the upregulation of voltage-gated T-type Ca^2+^ channels contributes to epileptogenesis via increases to both Ca^2+^ currents and neuronal burst firing (Becker et al., 2008), and similar Ca^2+^ influx through TRPC channels is suggested to contribute to hyperexcitability (Phelan et al., 2012). A missing link remains in the precise regulation of ACh neurotransmission that factors into the control of cell-specific signaling, in part by TRPC channels. The mAChR-induced depolarization promotes synchronous neuronal firing and seizures and involves activation of certain TRPC channels (Phelan et al., 2012; Carver and Shapiro, 2019). Pathophysiological modulation of mAChRs has been demonstrated to contribute to seizures in chronically epileptic rats (Zimmerman et al., 2008), and excessive stimulation of G_*q/*__11_-coupled mAChRs and PLC activation after TBI contribute to long-term pathophysiological and neurological deficits (Jiang et al., 1994; Lyeth et al., 1996). Furthermore, sustained increases in diacylglycerol (DAG) production at the neuronal membrane after TBI suggests involvement of lipid signaling pathways that contribute to the secondary injury cascade (Homayoun et al., 2000).

After TBI, the dentate gyrus (DG) has been shown to be diminished in its ability to regulate cortical input into the hippocampus, and this in turn results in increased CA3 network excitability and disruptive dysfunction of hippocampal information processing (Folweiler et al., 2018). Our previous work indicates that mAChR stimulation causes PLC-dependent activation of TRPC cation channels, contributing to aberrant hyperexcitability in the hippocampus (Carver and Shapiro, 2019). We hypothesize that TBI induces upregulation of TRPC channel transcription and protein expression, a mechanism that amplifies cholinergic-dependent hyperexcitability in both the hippocampus and cortex. We follow activity-dependent effects of TBI in the cortex as a reference for first-contact neurons to the trauma, and the hippocampus was studied to investigate network hyperexcitation that could be the precursor to epileptiform seizures. We describe this mechanism of hyperexcitability by investigation of TRPC channel-driven neuromodulation that may contribute to seizure susceptibility and further investigate the cortical–hippocampal axis of mAChR actions that engender brain hyperexcitability.

## Materials and Methods

### Animals

Wild-type adult male C57BL/6J mice (RRID: IMSR_JAX:000664) between 2 and 3 months of age were used for expression and behavioral experiments. Homozygous GCaMP6f mice [B6;129S-Gt(ROSA)26Sor^TM 95^.^1(*CAG–GCaMP*6*f*)*Hze*^/J; Jackson Laboratory Stock No. 024105, Jackson ImmunoResearch Laboratories, Inc., West Grove, PA, United States] were crossed with hemizygous Cre-CamKII [B6.Cg-Tg(Camk2a-cre)T29-1Stl/J; Jackson Laboratory Stock No. 005359, Jackson ImmunoResearch Laboratories, Inc.] mice to record Ca^2+^ signals in the brain. Ai14-tdTomato mice [B6;129S6-Gt(ROSA)26Sortm14(CAG-tdTomato)Hze/J; Jackson Laboratory Stock No. 007914, Jackson ImmunoResearch Laboratories, Inc.] and Fos2A-iCreER/TRAP2 mice [Fostm2.1(iCre/ERT2)Luo/J; Jackson Laboratory Stock No. 030323, Jackson ImmunoResearch Laboratories Inc.] were crossed in studies of neuronal activity. All mice were housed in an environmentally controlled animal facility with a 12-h light/dark cycle and had access to food and water *ad libitum*. Animals were cared for in compliance with the guidelines in the National Institutes of Health Guide for Care and Use of Laboratory Animals. All animal procedures were performed in a protocol approved by the Institutional Animal Care and Use Committee of University of Texas Health San Antonio and secondarily approved by Animal Care and Use Review Office of the Department of Defense.

### Controlled Cortical Impact Traumatic Brain Injury

The controlled cortical impact (CCI) TBI model was performed, as previously described by our group (Vigil et al., 2019). The CCI model causes a blunt TBI, with the skull remaining intact with minimal hematoma that normalizes 7 days postinjury (Hunt et al., 2009; Ren et al., 2013). This type of brain injury model most closely simulates the TBI experienced during blunt-head injuries from vehicular accidents or falls. Mice were anesthetized with isoflurane (3% induction, 1% maintenance) in 100% O_2_. Body temperature of 37°C was maintained using a temperature-controlled heated surgical table. The skull was fixed with ear bars into a stereotaxic frame. A small midline incision was made on the scalp using aseptic surgical techniques. Impact was delivered by a cylindrical probe of 5 mm diameter with an Impact One neurotrauma impactor (Leica Biosystems, IL, Buffalo Grove). A calibrated impact was delivered at 4.5 m/s to a depth of 1 mm and 0.5 s dwell time to the right parietal cortex [+1.8 mm medial–lateral (ML), −2.0 mm anterior–posterior (AP), and −1.0 mm dorsal–ventral (DV)]. Apnea episodes lasted between 10 and 60 s after impact. The scalp was then sutured closed, and mice were placed in a warming recirculation unit and monitored until fully awake and moving freely. Control littermate mice received anesthesia and sham surgery but did not receive impact. For consistency, the left hemisphere of both sham and TBI mice is designated as “contralateral” brain, and the right hemisphere is referred to as “ipsilateral,” despite no impact injury occurring in sham mice.

### Quantitative Real-Time PCR

Quantitative real-time PCR (qRT-PCR) was performed to quantify the messenger RNA (mRNA) levels of TRPC1/TRPC4/TRPC5 24 h and 7 days after CCI. Cortical and hippocampal samples were dissected and snap frozen in dry ice and stored at −80°C until analysis. Samples were homogenized in TRIzol buffer (Thermo Fisher Scientific, MA, Waltham) using an Ultra EZgrind tissue homogenizer (Denville Scientific, NJ, Swedesboro) at the lowest speed. After chloroform extraction, total RNA was precipitated and resuspended in nuclease-free water. RNA quality and quantity were tested for each sample using a spectrophotometer (NanoDrop). Complementary DNAs (cDNAs) were synthesized with SuperScript III first-strand (Invitrogen, Carlsbad, CA, United States) following the manufacturer’s protocol. Oligo(dT)_20_ (Invitrogen) was used for mRNA specific amplification, and 500 ng of total RNA was used in each reaction. For amplification of TRPC1, TRPC4, TRPC5, and the housekeeping gene hypoxanthine phosphoribosyl transferase (HPRT), the primers were as follows:

TRPC1: F-ACCTTTGCCCTCAAAGTGGT, R-GCCCAA AATAGAGCTGGTTG;
TRPC4: F-GGAATCATGGGACATGTGG, R-CGGAGG GAACTGAAGATGTTT;
TRPC5: F-GTGGATTCACGGAATACATCC, R-TTGCC AGGTAGAGGGAGTTC;
HPRT: F-ATACAGGCCAGACTTTGTTGGATT, R-TCAC TAATGACACAAACGTGATTCAA.

Quantitative real-time PCR reactions were performed in a 7900 HT Fast Real-Time PCR system (Life Technologies) using SYBR Green PCR master mix (Thermo Fisher Scientific, Waltham, MA, United States). The reaction begins with one step of 95°C for 10 min, followed by 40 cycles of 95°C for 15 s and 60°C for 1 min. The number of cycles needed to reach exhaustion of the reaction and the threshold cycle (CT) were determined, and the difference between template CT and housekeeping gene CT (ΔCT) was calculated and transformed in power of 2 (2^–Δ^ ^Δ^ ^*CT*^). Each sample was run in triplicate for each primer.

### Western Immunoblots

Regional brain samples or microdissected brain slices were collected 2 h, 2 days, or 7 days after sham surgery or TBI (ipsilateral and contralateral) and snap frozen in dry ice. Samples were homogenized on ice in a solution of radioimmunoprecipitation assay (RIPA) buffer (Thermo Fisher), protease inhibitor tablet and phosphatase inhibitor cocktail 2/3 (Sigma-Aldrich, MO, St Louis) using an Ultra EZgrind tissue homogenizer (Denville Scientific Inc.) at the lowest speed. Protein concentrations of each sample were determined using a Pierce BCA protein assay kit (Thermo Fisher). Protein samples (10–25 μg of total protein) were run in 10% precast polyacrylamide gels (Bio-Rad, Hercules, CA, United States) at 130 V for 1 h. Proteins were then transferred to polyvinylidene fluoride (PVDF) membrane with a Transblot SD semi-dry transfer system (Bio-Rad) at 15 V for 15 min. Membranes were blocked with 5% bovine serum albumin (BSA) and stained with rabbit polyclonal antibodies for TRPC1 (1:1,000, Alomone Labs #ACC-010, RRID:AB_2040234), TRPC4 (1:1,000, Alomone Labs #ACC-018, RRID:2040239), TRPC5 (1:250, Sigma-Aldrich #T0325, RRID:AB_262146), or muscarinic acetylcholine receptor type 1 (1:2,000, Millipore AB5164, RRID:AB_91713) for semiquantification of proteins. Immunoblot bands were acquired via horseradish peroxidase-conjugated secondary antibodies and enhanced chemilumiscence Western blotting detection reagents (GE Healthcare, IL, Chicago). In some brain tissues, a double band was observed with TRPC4 antibody around 120 kDa, which has been previously described in immunoblot, likely linked to binding of both TRPC4α and TRPC4β protein isoforms (Dalrymple et al., 2002). Quantification of bands after exposure in the linear range used ImageJ software (National Institutes of Health, Bethesda, MD, United States). Proteins were normalized to mouse monoclonal β-actin antibody (1:20,000, Sigma-Aldrich #A5316 clone AC-74, RRID:AB_476743) at 42 kDa as a loading control. Results are standardized to the contralateral averages for each sham or TBI mice group.

### Brain Slice Immunohistochemistry

Sham and TBI mice were anesthetized with isoflurane, and brains rapidly excised, sliced (100–200 μm), and fixed in paraformaldehyde [4% w/v in phosphate-buffered saline (PBS)] for 20 min. Fixed brains were washed 6× in PBS for 5 min. The remaining fixed sections were blocked in 8% donkey serum with 0.1% Triton-X and 0.1% Tween-20 in PBS. Parietal cortex or dorsal hippocampus brain sections (−1.3 to −2.3 mm posterior to bregma) were probed with a TRPC4 mouse monoclonal antibody (1:1,000, Rockland #200-301-G54, RRID:AB_2611251) at 1:500 dilution and fluorescein isothiocyanate (FITC) donkey antimouse IgG (1:250, Jackson ImmunoResearch #715-095-150, RRID:AB_2340792). Type 1 mAChR (M1R) were colabeled using a polyclonal rabbit antibody (1:500, Sigma-Aldrich, #AB5164, RRID:AB_91713) and secondarily labeled with rhodamine red donkey antirabbit IgG (1:250, Jackson ImmunoResearch#711-025-152, RRID:AB_2315776). After washing with PBS, sections were mounted in antifading reagent with 4′,6-diamidino-2-phenylindole (DAPI) and imaged on a Nikon Eclipse FN1 upright microscope with 20 × Plan Apo and 40 × oil objectives and swept-field confocal imaging in the DG, CA3, and CA1 subregions of the hippocampus and parietal cortex. Intensity ratios for TRPC immunoreactivity were obtained at a fixed exposure time of 500 ms in 10 MHz/14-bit readout mode.

### TRAP Neuronal Activity Assays

Targeted recombination in active populations (TRAP, see above) male mice were injected with 50 mg/kg 4-hydroxytamoxifen (4-OHT) to elicit cFos-labeling with td-Tomato (Guenther et al., 2013). 4-OHT was prepared by dissolution in 100% ethanol at 10 mg/ml concentration. After evaporation of ethanol, sunflower seed oil was added to a final concentration of 5 mg/ml. 4-OHT was administered by i.p. injection either 12 h prior to CCI-TBI or sham or 7 days after CCI-TBI or sham. Another cohort of mice received vehicle oil as a negative control, in which we detected no spontaneous td-Tomato fluorescence. At the conclusion of experiments, brain slices were acquired for stereology, as above. Brain slices were fixed and mounted with Vectashield containing DAPI to determine the total percentage of neurons per field of view in imaging. Images (20×) magnification (561 nm emission filter cube) were acquired for each slice and hemisphere, with at least four separate slices for each region per brain. The numbers of td-Tomato + parietal cortex layer 5 neurons and hippocampal principal neurons were quantified by a person who was blinded to the experimental group of the tissue. Individual mice each received an average count of td-Tomato + neurons for each region. Then, single mouse counts were averaged from five to six mice per group.

### Acetylcholinesterase Assay

To measure acetylcholinesterase (AChE) activity in localized brain regions, we used the Amplex Red Acetylcholinesterase Assay Kit (Thermo Fisher) (Santillo and Liu, 2015). This assay measures AChE concentration by a series of reactions starting with the production of choline from acetylcholine by AChE, which is then converted to betaine aldehyde with the byproduct H_2_O_2_. The H_2_O_2_ is then reacted with horseradish peroxidase to catalyze the conversion of Amplex red (10-acetyl-3,7-dihydroxyphenoxazine) into the fluorescent chemical resorufin, which can be quantified via fluorometry. Assays were performed using black 96-well plates with 200 μl reaction mixture per well. The AChE reaction cocktail final concentration was 100 μM Amplex Red, 1 U/ml horseradish peroxidase, 0.1 U/ml choline oxidase (*Alcaligenes* sp.), and 50 μM acetylcholine. Whole brain regions or microdissected slices were homogenized, and lysates were collected for protein as above but without phosphatase inhibitors in the RIPA buffer. Ten micrograms of total sample protein was diluted with reaction buffer (50 mM *Tris*–HCl, pH 8.0) to 100 μl total volume, followed by the addition of 100 μl of reaction cocktail. Fluorescence of resorufin (λ excitation/emission = 530 nm/590 nm) was measured with a microplate reader after incubation of 30 min at 25°C in the dark. The activity of the reaction buffer without protein sample was measured in triplicate, and the average value was subtracted as background. A standardized concentration–response curve of 0.1–200 μM of AChE was acquired in the assay, and (AChE) was determined for each sample as factored into the best-fit logistic curve of the standard.

### Brain Slice Ca^2+^ Imaging

Transverse slices (300 μm) of the hippocampus were cut with a vibratome (Thermo Scientific Microm HM650V) from Cre-CamK2^+^/GCamp6f^±^ mice. Mice were anesthetized with isoflurane, and the brains were excised and placed in artificial cerebrospinal fluid (ACSF) at 3.5°C composed of the following (in mM): 126 NaCl, 3 KCl, 0.5 CaCl_2_, 5 MgCl_2_, 26 NaHCO_3_, 1.25 NaH_2_PO_4_, 15 glucose, 0.3 kynurenic acid, with pH adjusted to 7.35–7.40, with 95% O_2_–5% CO_2_, and 305–315–mOsm/kg. Hippocampal slices were maintained in oxygenated ACSF at 30°C for 60 min, and experiments were performed at 28°C. Bath recording solution consisted of (in mM) 124 NaCl, 3 KCl, 1.5 MgCl_2_, 2.4 CaCl_2_, 1.25 Na_2_H_2_PO_4_, 26 NaHCO_3_, 15 glucose, 0.001 tetrodotoxin (TTX), and 0.05 hexamethonium-Cl. Neurons were visually identified with a Nikon FN-1 microscope equipped with a 40× water-immersion objective and infrared differential interference contrast. GCaMP6f fluorescence was obtained using a SOLA Light Engine Illumination source (Lumencor, OR, Beaverton) with an output of 3.5 W through a 3-mm diameter liquid light guide through a Nikon ET-GFP filter. Nikon Elements software was used for image acquisition through a QIClick CCD camera (QImaging, Teledyne Photometrics, AZ, Tucson) with a Uniblitz Model VMM-D1 shutter driver. The region of interest (ROI) used for the DG granule layer field was an area of 200 μm × 100 μm (0.16 μm/pixel), using four to six slices from each animal and four mice per condition. Baseline time series of the DG granule layer were acquired in the presence of bath solution perfusion for 2 min. Experimentally, slices were then perfused with either ACh (0.3 mM), englerin A (1 μM EA), M084 (10 μM), La^3+^ (10 μM), or a combination thereof dissolved in bath solution at a flowrate of 2 ml/min for 5 min. Images were acquired in time series with an exposure time of 200 ms at 2–10 fps at 12-bit rate and no binning. Time-series image stacks were processed with ImageJ. Images had the background subtracted using the mean of the 5% lowest pixel values. Ca^2+^ signal events were quantified for each cell as the change in fluorescence divided by the baseline fluorescence (ΔF/F_0_) after background subtraction. ROI signals were detrended before quantification to account for drift due to gradual photobleaching of the background. Cellular events were thresholded by signal intensity > 1.5× the root mean square noise for the 2-min baseline recording. Group cell fluorescence data of responsive cells (neurons with Ca^2+^ events) per field were averaged in comparison with cells from mice of sham control conditions.

### *In vivo* Electroencephalograms

To determine seizure frequency and record brain susceptibility to seizures, mice were implanted with electroencephalogram (EEG) electrodes 24 h after sham or TBI. A three-channel tethered EEG system was used. The electrode pedestal (P1 Technologies) was secured to the skull with three anchored dental screws and connected to a preamplifier with gain of 25. The three leads were inserted over the left frontal cortex, left parietal cortex, and depth electrode lead into the hippocampus. The Stellate Harmonie acquisition hardware and software were used to collect coincident video and EEG activity. EEGs were inspected for presence of epileptiform spikes and seizures (Keller et al., 2010).

### Chemical Kindling Seizure Susceptibility Assay

Brain kindling is the epileptogenic process resulting from low-level, subconvulsive stimuli to the brain by frequent, repeated exposure. Since the initial excitation is very minor, there is little to no overt presentation of seizure or network synchronicity. However, through repeated stimulation, the brain is altered to become more sensitive to the stimulus, resulting in a gradual induction of circuit hyperexcitability and spread of seizures over time that can be electrographically observed in conjunction with motor behavior. Seizure susceptibility was evaluated by chemical kindling the GABA_*A*_ receptor antagonist pentylenetetrazol (PTZ), and the progression of motor seizure activity was observed (Ferraro et al., 1999). Mice were injected with PTZ (35 mg/kg, s.c.) once per day and scored for motor behavioral seizures. Four stages of seizure response to PTZ were categorized: (1) a progressive decrease in activity and exploration until the animal came to rest in a crouched or prone position of hypoactivity; (2) myoclonic and jerking spasms characterized by brief focal seizures lasting 1 s or less; (3) generalized clonus characterized by sudden loss of upright posture, forelimb clonus, rearing, and autonomic signs; and (4) tonic–clonic seizure characterized by generalized seizure followed by tonic hindlimb extension. Latencies to focal (partial clonic), generalized (generalized clonic), and maximal (tonic–clonic) behavioral seizures were recorded and referenced by EEG recording of coincident electrical activity. Mice were monitored for 1 h; however, they were considered without seizure if there was no incidence of forelimb clonus behavior within 30 min postinjection. The initiation of daily PTZ administration began 24 h or 7 days after completion of sham or TBI surgeries.

### Experimental Design and Statistical Analysis

Group data are displayed as the mean ± standard error of the mean (SEM). Protein and mRNA expression samples were compared from group averages of ipsilateral vs. contralateral brain tissue. Relative quantifications of TBI sample mRNA were normalized to sham controls to derive fold change in mRNA as a result of injury. Each Ca^2+^ imaging experimental group data were collected from brain slices of at least three to four mice. Assessment of group data expected to be normally distributed was completed by the two-sample Kolmogorov–Smirnov test using control condition in relation to the empirical distribution function. To avoid oversampling bias in neurons with greater Ca^2+^ activity, cumulative probability distributions of Ca^2+^ fluorescence data were developed for using the same number of events per neuron. Control neurons were pooled between contralateral and ipsilateral sides upon displaying no statistical differences in the measures analyzed. Statistical comparisons of parametric measures including protein and mRNA expression, cFos neuron quantification, Ca^2+^ imaging, and AChE concentration assays were performed using an independent, two-tailed *t*-test, followed by Tukey’s honestly significant difference (HSD) test *post hoc*. For the chemoconvulsant kindling with PTZ, non-parametric data were analyzed with a Mann–Whitney *U* test for independent samples, and the Wilcoxon signed-rank test was applied for dependent samples in behavioral data. In comparisons in which contralateral and ipsilateral hemisphere tissues from sham animals were not statistically different, the data from both hemispheres were convolved in comparison to TBI samples. The criterion for statistical significance was *p* < 0.05 unless otherwise specified.

## Results

### TRPC1/TRPC4/TRPC5 Channels Are Differentially Regulated After CCI-TBI

**FIGURE 1:**
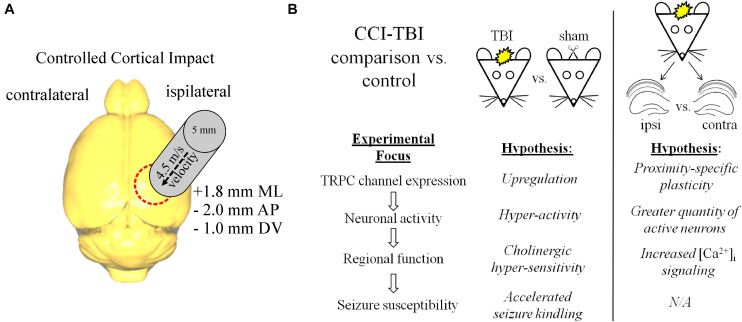
CCI-TBI diagram and analytical schema for comparative expression of TRPC channels. **(A)** Depicted is a dorsal view of the mouse brain and the area of impact of CCI-TBI over the ipsilateral cortex. Detailed approach can be found in “Materials and Methods” section. **(B)** Outlined are the design approaches and hypotheses for this study in the comparison of control vs. TBI and ipsilateral TBI vs. contralateral TBI investigations.

CCI-TBI was performed on adult male mice in this study (Figure 1A). TBI models often use the contralateral hemisphere as a “control” condition to compare with the injury-induced changes that are occurring at close proximity to the site of injury. However, the surviving neuronal/glial tissue on the contralateral side of the TBI brain often is far from normal, with ongoing posttraumatic reorganization and pathophysiology (Axelson et al., 2013; Pischiutta et al., 2018; Soni et al., 2019), and this contralateral tissue likely also contributes to epileptogenesis in the brain (Vigil et al., 2019). We sought to primarily compare sham control and TBI brain tissue in each of the experiments in which we tested regulation of TRPC channels (Figure 1B). In addition, we compared interhemispheric differences of TRPC channels in TBI animals in recognition that both hemispheres are likely differentially affected by injury. We chose to primarily investigate 7 days beyond the injury when both secondary cascades regulating neuronal excitability and synaptic plasticity are thought to commence (Hunt et al., 2009, 2013). CCI-TBI induces acute cortical and hippocampal damage and deficits that accrue across the first week after TBI (Saatman et al., 2006), and we hypothesized that TBI induces upregulation of TRPC1/TRPC4/TRPC5 channel expression in the brain.

We first compared TRPC1, TRPC4, and TRPC5 transcription between sham-surgery control mice and mice subjected to TBI. Seven days after the experimental procedure, microdissected hippocampus (DG, CA3, and CA1) and parietal cortex were collected and qRT-PCR performed. In TBI mice, we observed a >3-fold increase in both TRPC4 and TRPC5 mRNA from all regions tested in comparison to sham mice (Table 1, sham data not shown). In sham animals, there were no significant differences in mRNA levels between contralateral (left) and ipsilateral (right) brain tissue in the three major regions of the hippocampus or parietal cortex. Only the CA1 and CA3 regions from TBI mice exhibited upregulation of TRPC1 mRNA compared to shams. In TBI mice, DG and CA3 exhibited significantly greater TRPC4 mRNA in the ipsilateral region compared to the contralateral region, and CA3 exhibited greater TRPC5 mRNA ipsilaterally as well.

**TABLE 1:** Normalized fold change in TRPC1/4/5 mRNA (2^–ΔΔ^^*CT*^) in TBI mice compared to sham.

mRNA	Contralateral TBI	Ipsilateral TBI
**CTX**		
TRPC1	1.81 ± 1.05	1.13 ± 0.97
TRPC4	4.01 ± 0.66*	3.28 ± 0.65*
TRPC5	4.93 ± 1.16*	3.82 ± 0.79*
**DG**		
TRPC1	0.64 ± 0.38	0.43 ± 0.14
TRPC4	4.56 ± 0.11*	5.60 ± 0.47*^†^
TRPC5	6.52 ± 1.29*	6.10 ± 1.22*
**CA3**		
TRPC1	-0.07 ± 0.35	2.73 ± 0.36*^†^
TRPC4	3.72 ± 0.81*	4.52 ± 0.09*^†^
TRPC5	2.74 ± 0.59*	6.41 ± 0.54*^†^
**CA1**		
TRPC1	5.56 ± 0.31*	4.63 ± 0.49*
TRPC4	5.13 ± 0.27*	4.83 ± 0.25*
TRPC5	5.79 ± 0.43*	5.48 ± 0.51*

Summarized data for real-time PCR quantification (2^–ΔΔ^^CT^) of TRPC1, TRPC4, and TRPC5 mRNA of microdissected parietal cortex (CTX), dentate gyrus (DG), hippocampal CA3, and hippocampal CA1 regions 7 days after TBI procedure, normalized to sham tissue. All mRNA samples were first normalized to HPRT mRNA. n = 4 mice per group. Sham values not shown. *p < 0.05 vs. sham of same subregion. ^†^p < 0.05 vs. contralateral hemisphere of same subregion with unpaired two-tailed t-test. Data represent the mean ± SEM.

**FIGURE 2:**
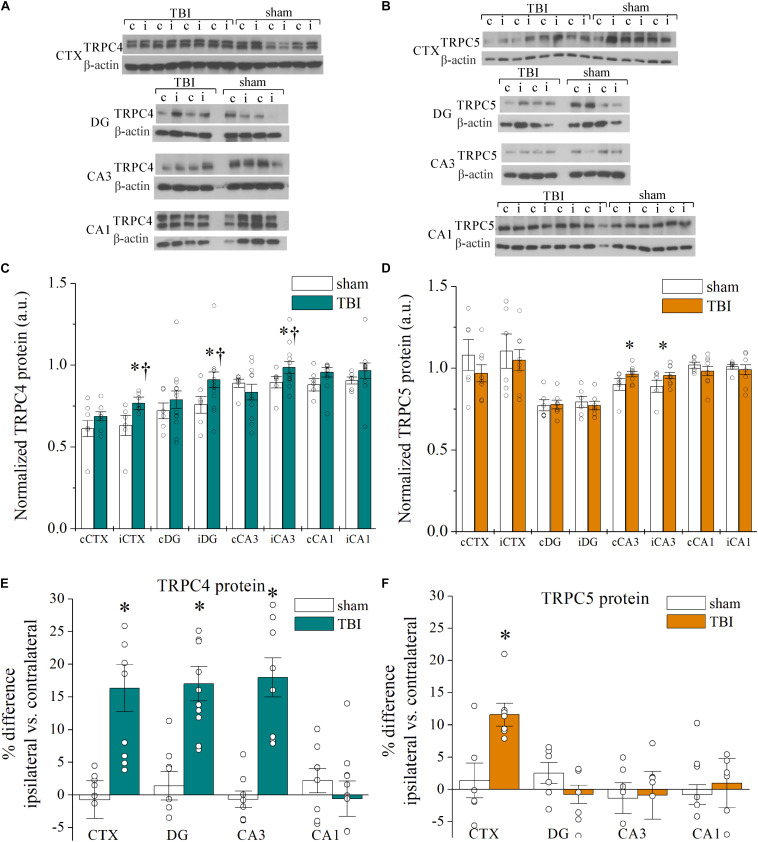
Cell-type specific TRPC1, TRPC4, and TRPC5 channel upregulation in the hippocampus and cortex after CCI-TBI. **(A,B)** Representative Western immunoblots using **(A)** TRPC4 (∼120 kDa) and **(B)** TRPC5 (∼110 kDa) antibodies in sham and TBI cortex and hippocampus. Blots were normalized to β-actin protein (42 kDa) as loading control. **(C,D)** Summarized data for Western blot quantification of TRPC4 (**C**, *n* = 7–13 animals per group) and TRPC5 (**D**, *n* = 5–7 animals per group) from microdissected brain regions in mice 7 days after TBI. **(E,F)** Shown are summarized plots of percent difference in TRPC4 **(E)** and TRPC5 **(F)** protein between ipsilateral and contralateral hemispheres of microdissected regions from data in Panels **(C,D)**. All data bars represent the mean ± SEM. *p < 0.05 vs. sham of same subregion. ^†^*p* < 0.05 vs. contralateral hemisphere of same subregion.

To determine if the upregulation of mRNA correlated with changes in channel protein after TBI, we analyzed Western immunoblots of TRPC channels in brain samples collected 7 days after sham and TBI (Figures 2A–F and Table 2). Ipsilateral DG (*p* = 0.030, *n* = 6–12), ipsilateral CA3 (*p* = 0.041, *n* = 6–12), and ipsilateral cortex (*p* = 0.016, *n* = 6–8) from TBI mice exhibited significantly greater mean TRPC4 protein expression than the equivalent regions from sham mice (Figure 2C). In some cases, protein immunoblots exhibited a double band upon TRPC4 binding, which may be a demarcation of the TRPC4α and TRPC4β isoforms (Mery et al., 2001; Dalrymple et al., 2002). We also found mean TRPC5 protein expression from contralateral CA3 (*p* = 0.028, *n* = 6–8) and ipsilateral CA3 (*p* = 0.041, *n* = 6–8) of TBI mice to be significantly greater than the sham group (Figure 2D).

**TABLE 2:** Normalized TRPC4/TRPC5 protein in 7-day sham and TBI mice.

Region/protein	Contralateral sham [*n*]	Ipsilateral sham [*n*]	Contralateral TBI [*n*]	Ipsilateral TBI [*n*]
CTX/TRPC4	0.61 ± 0.05 [6]	0.63 ± 0.06 [6]	0.69 ± 0.03 [9]	0.77 ± 0.03** [8]
CTX/TRPC5	1.08 ± 0.09 [6]	1.10 ± 0.10 [6]	0.97 ± 0.05 [8]	1.05 ± 0.06 [8]
DG/TRPC4	0.72 ± 0.05 [6]	0.78 ± 0.06 [6]	0.79 ± 0.05 [12]	0.91 ± 0.05* [12]
DG/TRPC5	0.77 ± 0.03 [6]	0.79 ± 0.03 [6]	0.78 ± 0.03 [7]	0.77 ± 0.03 [7]
CA3/TRPC4	0.89 ± 0.03 [6]	0.89 ± 0.04 [6]	0.83 ± 0.05 [12]	0.99 ± 0.04* [12]
CA3/TRPC5	0.90 ± 0.04 [6]	0.89 ± 0.04 [6]	0.96 ± 0.02* [7]	0.95 ± 0.02* [7]
CA1/TRPC4	0.88 ± 0.04 [6]	0.91 ± 0.02 [6]	0.96 ± 0.03 [12]	0.96 ± 0.05 [12]
CA1/TRPC5	1.02 ± 0.02 [6]	1.01 ± 0.01[6]	0.98 ± 0.03 [7]	0.99 ± 0.04 [7]

Protein values are normalized to β-actin and are listed as arbitrary units. Ten micrograms of total protein was loaded for each sample. Sample size n is listed in brackets beside each group and region. Data represent mean ± SEM. *p < 0.05 vs. sham of same group, **p < 0.01 vs. sham of same group, with unpaired two-tailed t-test.

The interhemisphere comparisons of protein in TBI mice are also of interest, due to established observation of differential, localized induction of excitability in the hippocampus and cortex (Hunt et al., 2010; Gao et al., 2011; Vigil et al., 2019). The ipsilateral cortex (*p* = 0.016), DG (*p* = 0.016), and CA3 (*p* = 0.020) subregions from TBI mice exhibited significant increases in mean TRPC4 protein compared to the contralateral tissue from TBI mice (Figure 2C). We analyzed the percent difference in TRPC4 and TRPC5 protein levels between contralateral and ipsilateral regions within each mouse and compared the relative change between sham and TBI mice. The disparity between ipsilateral and contralateral TRPC4 was significantly greater in the cortex (16.3 ± 3.5% increase, *p* = 0.016), DG (17.0 ± 2.5% increase, *p* = 0.007), and CA3 (18.1 ± 3.0% increase, *p* = 0.002) regions as compared to shams, but this was not the case for CA1 (−0.5 ± 2.7% change, *p* = 0.216; Figure 2E). Individual TBI mice demonstrated increased TRPC5 within the ipsilateral cortex when compared to their contralateral cortex (11.6 ± 1.8% increase, *p* = 0.015 vs. sham), but no TRPC5 differences were present in hippocampus (Figure 2F). Overall, the upregulation of protein expression of TRPC4/TRPC5 in 7 day post-TBI mice was consistent with the mRNA expressional data we acquired. However, in comparison of the control and TBI mice, the detected mRNA fold change in expression was substantially larger than the fold change in the protein measured. Assays of differential tracking to the membrane and protein turnover time would be required to parse out reasons for these differences, as is often the case.

Despite no significant differences in TRPC4 or TRPC5 protein expression within CA1, TRPC1 protein was elevated in the CA1 of TBI mice compared to sham CA1 (*p* = 0.021, *n* = 6–8), consistent with the mRNA findings (Supplementary Figure 1A). On the other hand, TRPC1 expression in the CA3 region was not significantly different between sham mice and TBI mice (*p* = 0.487). Therefore, TRPC1/TRPC4/TRPC5-containing channels in the CA1 may have a specialized modulatory role compared to TRPC channels in other hippocampal subregions. To determine if the changes in expression of TRPC channels are dependent on time post-TBI, we then measured TRPC4/TRPC5 protein expression from brains collected 14 days after TBI (Supplementary Figure 1B). TBI mice displayed further elevated TRPC4 protein expression in the cortex and CA3 in 14-day samples, beyond that of mice 7 days post-TBI. There were no significant differences in the expression of TRPC4 or TRPC5 between contralateral and ipsilateral tissues after 14 days.

To evaluate TRPC4 channel expression on a cellular basis, we performed immunohistochemistry on hippocampal slices, in which the neuronal architecture remains intact (Supplementary Figure 2). We detected TRPC4 expression that was mostly confined to the cell bodies of the principal neurons. Given the established link between cholingeric stimulation via G_*q/*__11_-coupled mAChRs and activation of TRPC channels in the brain, and the emerging theme of colocalization of signaling proteins into microdomains (Yan et al., 2009; Teles-Grilo Ruivo and Mellor, 2013), we also evaluated the subcellular localization of M_1_Rs in these experiments. We found M_1_Rs to be colocalized with TRPC4 to the soma and to the dendritic areas of the principal neurons of the hippocampus. These immunostaining data suggest differential expression of TRPC channel subtypes in cell type and in compartmental localization. The ramifications of such results are elaborated in the discussion below.

### Posttraumatic Effects of Neuronal Hyperexcitability

**FIGURE 3:**
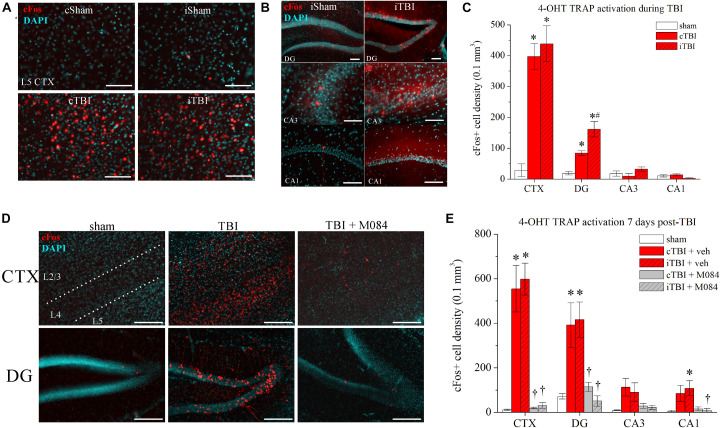
Surges in neuronal activity following CCI-TBI are TRPC4/TRPC5-mediated. **(A)** Representative images of parietal cortex from sham and TBI TRAP mice that were administered 4-OHT at *t* = 12 h before procedure. Prefix “*c*” denotes contralateral, prefix “*i*” denotes ipsilateral. Red = cFos-tdTomato; blue = DAPI. **(B)** Representative images of hippocampal subregions from sham and TBI TRAP mice that were administered 4-OHT at *t* = 12 h before procedure. **(C)** Summarized quantification of cFos+ neuron density (neurons/0.1 mm^3^) in sham and TBI TRAP mice activated at the time of TBI, as in Panels **(A,B)**. **(D)** Representative images taken from sham mice, TBI mice, and TBI mice also administered M084 (10 mg/kg) (TBI + M084) that were administered 4-OHT *t* = 7 days after procedure. **(E)** Summarized quantification of cFos+ neurons in sham, TBI, and TBI + M084 mice 7 days after procedure, as in Panel **(D)**. All data bars represent the mean ± SEM. *p < 0.05 vs. sham. ^#^*p* < 0.05 vs. TBI cDG. ^†^*p* < 0.05 vs. TBI of same region. Scale bars: 100 μm.

Traumatic brain injury promotes a host of pathological alterations that include brain swelling, inflammation, cellular damage, neuronal death (Anderson et al., 2005), and increased neuron hyperexcitability, the latter being key to the generation of epileptiform seizures (Santhakumar et al., 2001). Traditional studies for the induction of neuronal hyperactivity are usually confined by a single snapshot at the time of animal sacrifice to study histological factors including cFos immunoreactivity. This is because as an immediate/early gene, cFos is only transiently active for 2–3 h after a hyperexcitatory event, beyond which time the signal can no longer be detected (Yang et al., 1994; Peng and Houser, 2005; Barros et al., 2015). In order to quantify the time course of cFos activation in response to TBI-induced hyperactivity, we used mice containing the transgene construct for cFos-TRAP for targeted recombination in active populations (Guenther et al., 2013; Figure 3). In TRAP mice, neurons expressing cFos/CreER^*T*2^ that are conditionally activated with 4-OHT produce and maintain the presence of a constitutively-active td-Tomato fluorescent marker, thereby enabling the detection of overall neuronal activity within a short window of time *in vivo* (Figure 3A). Therefore, we are able to quantify the cell-type specific fraction of neurons in a given volume of tissue that are overtly active in response to TBI. Initially, we administered 4-OHT to mice 30 min before the sham/TBI procedure. The surgery recovery rates of injected animals were very low, and a majority of mice died, precluding our ability to test neuronal effects of the TBI with Cre recombination. A lower dose 30 min before surgery did not improve survival outcome. The 4-OHT compound is very potent in promoting recombination (EC_50_ = 7.6 ng/ml) and is reported to have a half-life of 15.8 h in mice (Valny et al., 2016; Jahn et al., 2018). We elected to administer 50 mg/kg 4-OHT to TRAP mice 12 h prior to performing TBI or sham surgery (Figures 3A–C). Although 4-OHT levels are trailing at the time of the trauma event 12 h after administration, there is sufficient non-metabolized 4-OHT to affect recombination (minimal effective concentration is 4.5 ± 1.3 mg/kg; Valny et al., 2016). Furthermore, the sham control animals injected with 4-OHT served as the baseline level of activity in the procedure, enabling the ability to assess if TBI caused excitability beyond the effect of surgery itself.

To observe the actions at the site of injury at the parietal cortex, we focused on differences specific to layer 5 neurons, which have been reported to exhibit spontaneous increase in firing after injury (Johnstone et al., 2013, 2014), and any further data reporting of cellular-level cortex was focused on layer 5 neurons. In sham mice, basal activity of cortical neurons was relatively low, with only one or two td-Tomato-positive neurons per field (Figure 3C). In contrast, there were profound increases in the numbers of td-Tomato cortical neurons from TBI mice, indicative of robust cFos activity compared to the basal quiescence in sham animals [contralateral CTX vs. sham: *t*_(__6__)_ = 7.85, *p* = 0.0002; ipsilateral CTX vs. sham: *t*_(__6__)_ = 6.63, *p* = 0.0006; *n* = 4 mice/group]. Fractions of cFos+ neurons are reported as the normalized cell density measurement in a volume of 0.1 mm^3^. Raw numbers of neurons that were counted per slice image field are presented in Table 3. Due to the pervasive spread of the td-Tomato fluorophore within the cell, TRAP-activated neurons showed dendritic processes that were illuminated as well. Higher magnification images confirmed that the active layer 5 cells were pyramidal neurons, based on their morphology visualized by td-Tomato (Supplementary Figure 3A). The DGGCs also had a notable increase in the fraction of cFos+ neurons bilaterally in TBI mice [contralateral vs. sham: *t*_(__6__)_ = 6.73, *p* = 0.0005; ipsilateral vs. sham: *t*_(__6__)_ = 5.55, *p* = 0.0014; *n* = 4 mice/group] (Figure 3B). In particular, the DGGCs that were cFos+ after TBI were mature neurons with full dendritic arborizations in the molecular layer (Supplementary Figure 3B).

**TABLE 3:** cFos+ neuron counts in regional image field of view for sham and TBI TRAP mice 7 days postprocedure.

Region	sham	cTBI + veh	iTBI + veh	cTBI + M084	iTBI + M084
CTX	6.6 ± 1.6	322.0 ± 60.5*	350.4 ± 41.6*	11.1 ± 2.1^†^	17.8 ± 7.3^†^
DG	6.4 ± 1.1	34.7 ± 10.3*	37.3 ± 7.1*	10.3 ± 1.9^†^	4.5 ± 2.1^†^
CA3	0.8 ± 0.2	9.3 ± 3.3	7.4 ± 3.5	2.3 ± 0.9	1.8 ± 0.7
CA1	0.3 ± 0.2	4.4 ± 1.9	5.6 ± 1.8	0.8 ± 0.4	0.4 ± 0.3

cFos+ neurons are principal neurons positively fluorescent with tdTomato. Counts from each region were collected from four to five slices per animal and five to seven animals per condition. Data represent mean ± SEM. *p < 0.05 vs. sham of same region, ^†^p < 0.05 vs. TBI + veh of same region, with unpaired two-tailed t-test.

We wished to determine if *in vivo* blockade of TRPC4/TRPC5 channels using the antagonist M084 (Zhu et al., 2015) would decrease TBI-dependent neuronal activity. In a time-delayed study, we administered 4-OHT to TRAP mice at 7 days after sham or TBI (Figures 3D–F). This timing coincides with the period of significant TRPC4 upregulation after TBI (Figure 2). A cohort was administered M084 (10 mg/kg) twice daily for 7 days after TBI to maintain a steady-state blockade of TRPC4/TRPC5 channels (Yang et al., 2015). In anticipation of this injection regimen, the parallel cohorts of sham and TBI mice were administered vehicle on the same schedule. In visualization of cFos-active neurons, the 7-day sham hippocampus and cortex had nominal cFos activity, similar to the previous group of shams in Figures 3A–C. Sham animals typically displayed 0 or 1 active neurons in each regional slices of CA3 and CA1, whereas the 7-day TRAP TBI mice exhibited a small increase in cFos activation in CA1 and CA3 pyramidal neurons (Figure 3D). However, we observed overt increases to cFos+ principal neuron populations in both the cortex and DG of untreated TBI mice (Figure 3E). The percentage of cFos+ DGGCs increased from 0.7 ± 0.1% across all shams to 4.1 ± 1.5% in contralateral TBI tissue and 4.5 ± 0.8% in ipsilateral TBI tissue. Conversely, mice treated with M084 exhibited sparse cFos activation, as cFos+ neurons were significantly fewer in number than in untreated TBI mice (Figure 3E). In those TBI mice treated with M084, the percentage of DGGCs responding during the window of activation was 1.2 ± 0.2% in contralateral DG and 0.5 ± 0.2% in ipsilateral DG. Therefore, the latent neuronal activity induced by TBI was diminished by the blockade of TRPC4/TRPC5 channels in the DG and cortex.

### Modulation of TRPC Generates Robust Ca^2+^ Influx and Promotes Neuronal Hyperexcitability

**FIGURE 4:**
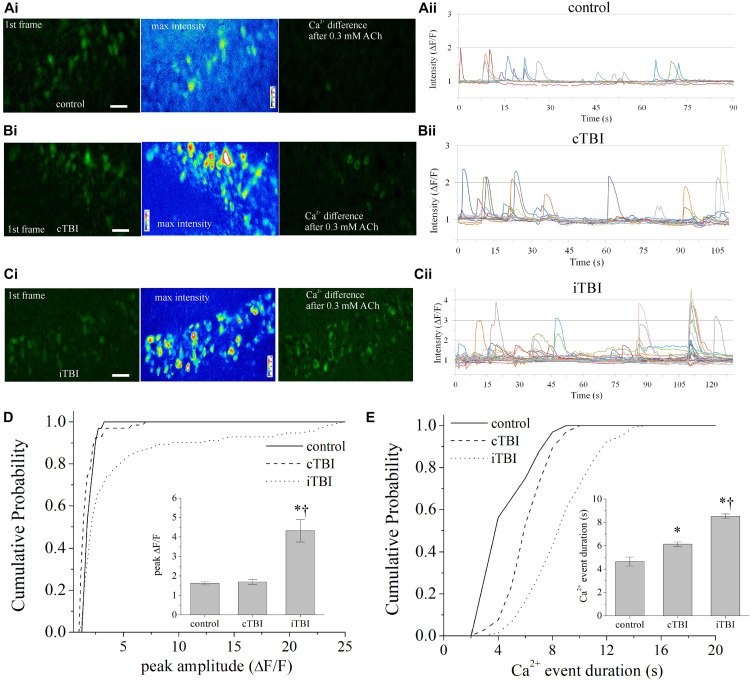
TRPC-dependent cholinergic Ca^2+^ influx in brain slice subsequent to CCI-TBI. **(A–C)** Representative GCaMP6f Ca^2+^ activity (green) images from the DGGC layer of live brain slices for **(Ai)** control sham mice, **(Bi)** contralateral DGGCs to TBI (cTBI), and **(Ci)** ipsilateral DGGCs to TBI (iTBI) 7 days after procedure. Left image: showing the first frame of recording; middle image, the maximum intensity value for each pixel of Ca^2+^ imaging after application of 0.3 mM ACh, shown with 16-color heat map; right image: the composite difference in fluorescence intensity in comparing all frames to the first frame. Higher green fluorescence denotes greater Ca^2+^ influx activity. **(Aii–Cii)** GCaMP6f fluorescence intensity (ΔF/F) of DGGCs over time of bath application of 0.3 mM ACh. **(Aii)** Control sham, **(Bii)** contralateral DGGC after TBI, and **(Cii)** ipsilateral DGGC after TBI. Experiments were carried out in the presence of hexamethonium chloride (50 μM) and TTX (1 μM). **(D)** Cumulative probability distribution of the peak amplitude of GCaMP6f fluorescence (ΔF/F) for each DGGC from control, cTBI, and iTBI slices. **(D)** (Inset) Summarized mean data of peak fluorescence intensity. **(E)** Cumulative probability distribution of the Ca^2+^ event duration (in seconds) for each DGGC from control, cTBI, and iTBI slices. **(E)** (Inset) Summarized mean data of Ca^2+^ event duration. All data bars represent the mean ± SEM. *p < 0.05 vs. control, ^†^*p* < 0.05 vs. cTBI. Scale bars: 20 μm, 40× magnification.

At 8–12 weeks after injury, a localized and recurrent excitatory network forms in the DG (Hunt et al., 2010), with origins of regulatory disruptions promoting hyperexcitation within days post-TBI (Neuberger et al., 2019). Upon observation of region-specific TRPC upregulation in neurons, we hypothesized that such plasticity promotes hyperexcitatory activity early in the posttraumatic hippocampus. We previously described that G_*q*__/__11_-coupled M_1_Rs evoke persistent Ca^2+^ influx, largely through TRPC channels, which contribute to DGGC excitability (Carver and Shapiro, 2019). To further assess the postinjury effect of TRPC channel upregulation on neurons, we investigated ACh-induced changes in intracellular [Ca^2+^] in brain slice neurons *ex vivo* (Figure 4) using GCaMP6f transgenic mice (Éltes et al., 2019). We hypothesized that DGGCs from TBI mice exhibit greater sensitivity to ACh than sham controls. We did not delineate the cytoplasmic Ca^2+^ increases between IP_3_-mediated release and TRPC channel conductance. However, our previous work demonstrates that TRPC channels contribute the majority of increases to intracellular Ca^2+^ due to mAChR stimulation (Carver and Shapiro, 2019). Sensitivity was tested by bath perfusion of ACh to the slice for 5 min during Ca^2+^ imaging to quantify peak amplitude of GCaMP6f fluorescence per cell, duration of Ca^2+^ increase, and the field frequency of events (Figure 4). Hexamethonium chloride (50 μM) was included in the perfusion solution to block nAChRs.

In sham animal recordings, single-cell Ca^2+^ transient events had an average duration of 4.6 ± 0.4 s and a 10–90% rise time of 1.1 ± 0.1 s, as measured by GCamp6f fluorescence. Bath application of 0.3 mM ACh increased the frequency of intracellular [Ca^2+^] events in DGGCs from TBI mice but not in sham control DGGCs (Figure 4). The [Ca^2+^] events persisted in the presence of 1 μM TTX. From TBI mice, DGGCs from the ipsilateral hemisphere demonstrated significantly greater numbers of ACh-responsive neurons per field than DGGCs from the contralateral hemisphere (Supplementary Figure 4A). During perfusion of ACh, the contralateral hemisphere of TBI mice displayed an average of 2.6 ± 0.2 events/neuron, and the ipsilateral hemisphere of TBI mice displayed 4.3 ± 0.2 events/neuron [*t*_(__161__)_ = 4.90, *p* < 0.0001], whereas sham control tissue averaged 1.8 ± 0.3 events/neuron. Area under the curve values of [Ca^2+^] events (peak amplitude × event duration) were greater in both contralateral and ipsilateral TBI compared to control, and under the curve from the ipsilateral was greater than that from the contralateral hemisphere of TBI mice (Supplementary Figure 4B). The peak amplitude of events detected by GCamp6f fluorescence during ACh was 1.70 ± 0.12 ΔF/F in DGGCs from the TBI contralateral hemisphere and 4.31 ± 0.58 ΔF/F in ipsilateral DGGCs [*t*_(__209__)_ = 3.09, *p* = 0.0025, Figure 4D and Supplementary Figure 4C]. Despite the increased variance in the peak amplitude of responses from neurons from the ipsilateral hemisphere, the mean duration of ipsilateral [Ca^2+^] events (8.51 ± 0.20 s) was consistently and significantly longer than in contralateral DGGCs (6.12 ± 0.19 s) and groups had similar variance [*t*_(__209__)_ = 7.39, *p* < 0.0001, Figure 4E and Supplementary Figure 4D].

**FIGURE 5:**
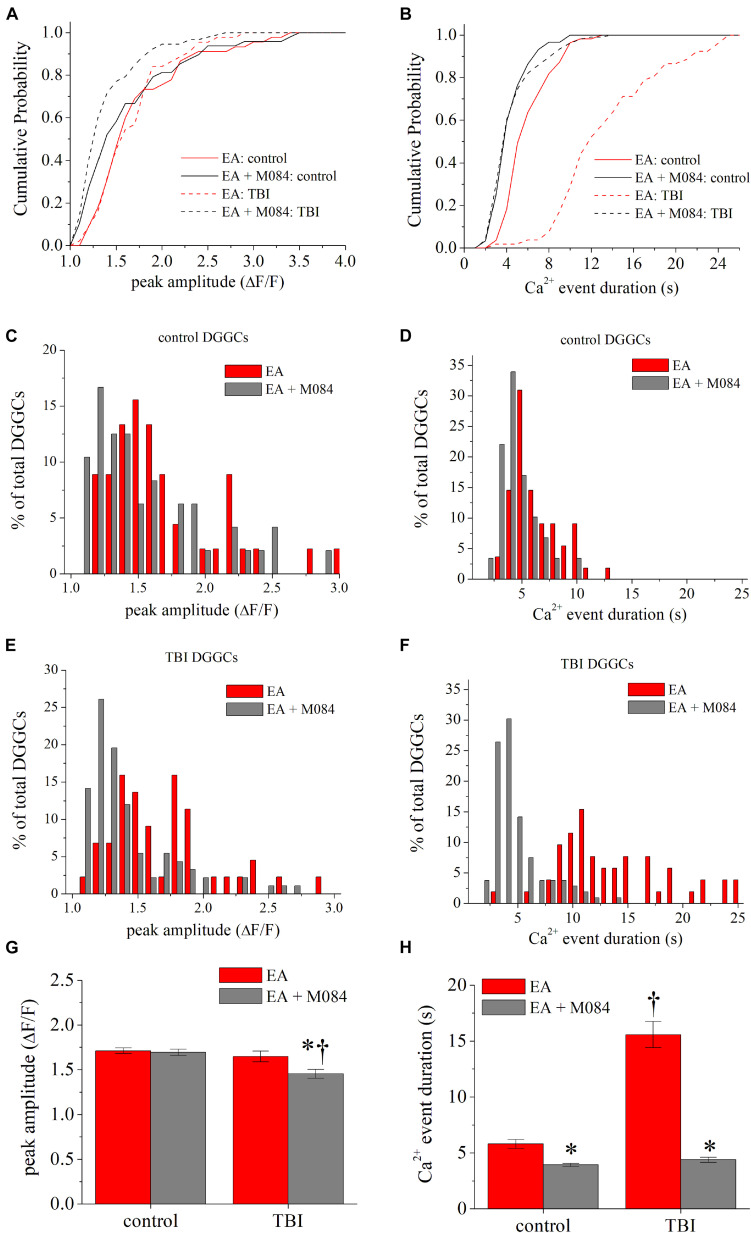
TRPC4/TRPC5 channel activation artificially prolongs Ca^2+^ influx in DGGCs after CCI-TBI. **(A)** Cumulative probability distribution of the peak amplitude of GCaMP6f fluorescence (ΔF/F) for each DGGC from sham (control) and iTBI slices during EA (1 μM, red) or EA + M084 (10 μM, gray) application. **(B)** Cumulative probability distribution of the Ca^2+^ influx duration (in seconds) for each DGGC from control and iTBI slices during EA or EA + M084 application. **(C,D)** Histogram population distribution of control DGGC Ca^2+^ influx events according to peak amplitude **(C)** and Ca^2+^ event duration **(D)**. **(E,F)** Histogram population distribution of iTBI DGGC Ca^2+^ influx events according to peak amplitude **(E)** and Ca^2+^ event duration **(F)**. **(G)** Summarized means of peak fluorescence from data as in Panel **(A)**. **(H)** Summarized means of Ca^2+^ influx duration from data as in Panel **(B)**. Red = EA alone, gray = EA + M084. All data bars represent the mean ± SEM. *p < 0.05 vs. EA alone from same procedure condition, ^†^*p* < 0.05 vs. control of same drug condition.

To further investigate TRPC channel activity after TBI, we used EA, a potent agonist of TRPC4/TRPC5 channels (Akbulut et al., 2015; Grant et al., 2019). We hypothesized that direct targeting of TRPC4-containing channels elicits robust Ca^2+^ signals, similar to those from cholinergic activity described above. We measured Ca^2+^ signals in the absence and presence of a saturating concentration of EA (1 μM) in ipsilateral DGGCs from sham and TBI mice. In TBI mice, [Ca^2+^] peak amplitudes from bath application of EA (1.66 ± 0.06 ΔF/F) was not significantly altered from the baseline control (1.62 ± 0.08 ΔF/F) [KS test: D_(__74__)_ = 0.17, *p* = 0.63]. However, when M084 (10 μM) was added, the Ca^2+^ signals decreased significantly in amplitude [1.44 ± 0.05 ΔF/F; KS test: D_(__151__)_ = 0.42, *p* < 0.001; Figures 5A,E,G]. In sham mice, [Ca^2+^] amplitudes were not significantly different between EA (1.71 ± 0.01 ΔF/F) and EA + M084 (1.69 ± 0.02 ΔF/F) conditions [KS test: D_(96)_ = 0.27, *p* = 0.07; Figures 5C,G], but M084 significantly reduced the duration of the Ca^2+^ signals in sham control DGGCs [KS test: D_(__116__)_ = 0.448, *p* < 0.001; Figures 5D,H]. Interestingly, EA application alone resulted in considerably prolonged duration of intracellular Ca^2+^ increase in TBI DGGCs (15.4 ± 1.1 s) (Figures 5B,F,H). Application of M084 to slices attenuated the duration of the Ca^2+^ signals to near baseline activity (4.4 ± 0.2 s) with a cumulative distribution significantly different than the distribution of events during EA alone [KS test: D_(__150__)_ = 0.83, *p* < 0.001], suggesting mediation of TRPC4/TRPC5-containing channels in this prolonged activity of [Ca^2+^] events. Overall, these data support the role of TRPC4/TRPC5-containing channels to elicit strong Ca^2+^ influx within DGGCs after TBI.

### AChE Is Significantly Reduced After Trauma and Remains Low in the Hippocampus and Cortex

**FIGURE 6:**
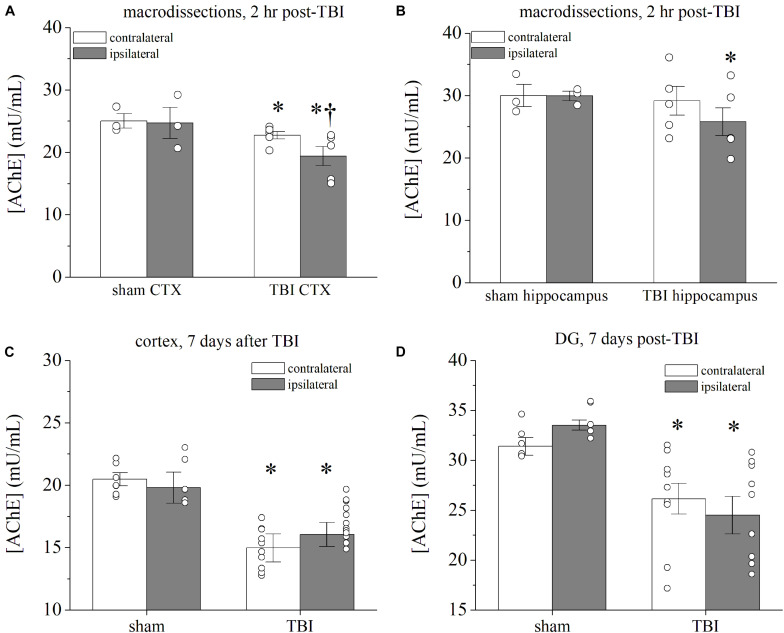
Prolonged decrease in AChE within hippocampus and cortex after CCI-TBI. **(A,B)** Summarized data for acetylcholinesterase (AChE) assay of macrodissected brains [CTX = cortex in panel **(A)**, HC = hippocampus in panel **(B)**] 2 h after TBI or sham procedure. The assay measures Amplex Red fluorescence of acetylcholinesterase enzymatic activity. Bars represent concentration of acetylcholinesterase present in protein homogenates in mU/ml. Ipsilateral cortex and hippocampus of TBI mice exhibit significant decrease in AChE compared with contralateral tissue. **(C,D)** Summarized data for AChE assay in panel **(C)** DG and **(D)** cortex 7 days after TBI. Whereas ipsilateral and contralateral AChE was not significantly different, TBI tissue exhibited significantly less AChE activity than sham controls. All data bars represent the mean ± SEM. *p < 0.05 vs. sham tissue, ^†^*p* < 0.05 vs. contralateral tissue. *n* = 5–12 mice per group.

Previous research has suggested that increased synaptic storage of ACh (via increase in VAChT) may occur in compensatory response to TBI in order to increase efficiency of cholinergic neurotransmission (Ciallella et al., 1998; Shao et al., 1999). Likewise, in support of altered cholinergic activity, AChE is decreased in the hippocampus 2 and 24 h after CCI injury, and the motor cortex exhibits a decrease in AChE 24 h after injury (Donat et al., 2007). Furthermore, patients with TBI have significantly lower chronic AChE activity in the cortex (Ostberg et al., 2011), as measured by functional imaging (Ostberg et al., 2011, 2018). However, there has been no conclusive investigation of AChE at intervals further from the time of occurrence of the TBI. Given that TRPC4-containing channels are upregulated 7 days after TBI, we hypothesized that cholinergic activity is likewise facilitated by diminished AChE in posttraumatic brain. We assayed AChE activity in the brains of sham and TBI mice in order to determine if higher levels of extracellular ACh may affect excitatory responses. Moreover, we opted for an enzymatic analytical approach to achieve high sensitivity using the Amplex-Red assay of AChE that employs enzymatic generation of H_2_O_2_ (see “Materials and Methods” section). As a control, saturating concentration of the AChE antagonist neostigmine (50 nM) applied to the same tissue samples attenuated the signal intensity of Amplex-Red by 94.0 ± 0.6% (*n* = 16 samples). Detection of Amplex-Red direct interaction with 1–10 μM H_2_O_2_ also confirmed that the assay was consistent in each reading and was used to determine the signal ceiling for calibration. We first probed regionally isolated tissues collected 2 h after TBI (Figures 6A,B). TBI parietal cortex exhibited bilateral decreases in AChE levels compared to the cortex from control animals (Figure 6A). In the whole hippocampus samples, AChE was decreased in the ipsilateral hemisphere of the TBI (Figure 6B). In examination of brains at 7 days post-TBI, we observed even larger deficits of AChE in both ipsilateral and contralateral micro-dissected tissue as compared to samples from sham control mice (Figures 6C,D). In the microdissected parietal cortex, AChE was reduced by 20.8 ± 6.7 and 18.3 ± 5.6% in the contralateral (*p* = 0.025) and ipsilateral (*p* = 0.018) hemispheres of TBI mice, respectively (Figure 6C). AChE was reduced 18.6 ± 5.4 and 20.1 ± 7.1% in the contralateral (*p* = 0.019) and ipsilateral (*p* = 0.022) DG of TBI mice, respectively (Figure 6D). In the CA3 region, AChE was reduced 13.3 ± 0.05 and 18.7 ± 0.04% in the contralateral (*p* = 0.04) and ipsilateral (*p* = 0.008) tissues, respectively. In contrast, the CA1 only exhibited minor fluctuations in AChE concentrations, as the reduction in contralateral (−4.4 ± 1.2% reduction, *p* = 0.062) and ipsilateral hemispheres (1.3 ± 2.0% increase, *p* = 0.565) of TBI mice were not significantly different from sham. Overall, decreased AChE concentrations in the hippocampus and cortex after TBI suggest a reprioritization of cholinergic signaling as a result of injury.

### TBI Facilitates TRPC-Dependent Seizure Susceptibility

**FIGURE 7:**
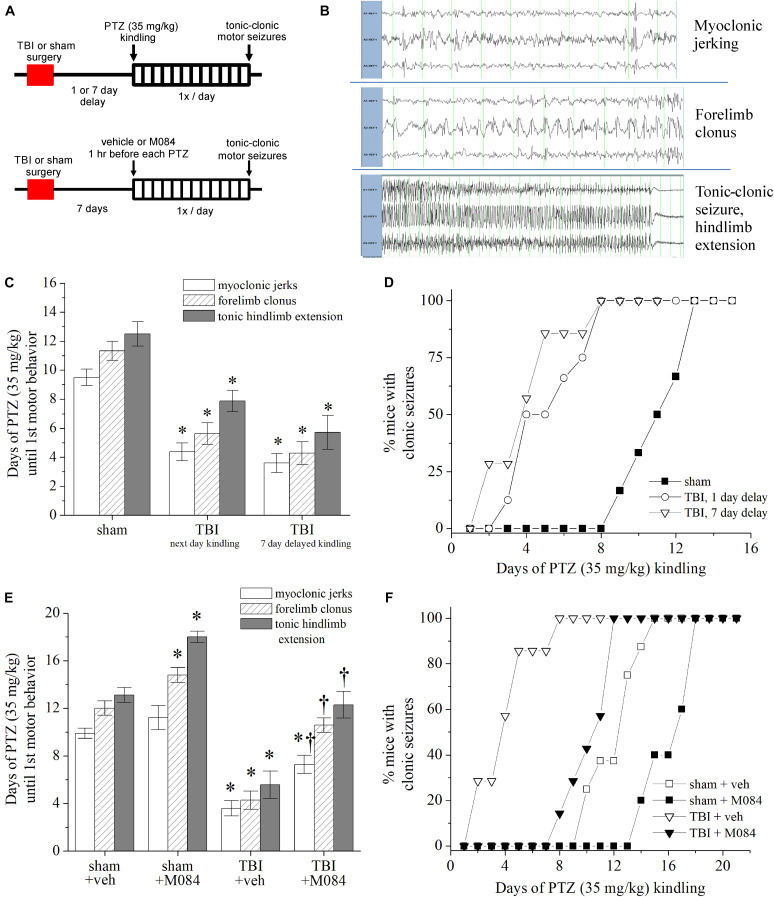
Contribution of TRPC channels to posttrauma seizure susceptibility in mice. **(A)** Design schematic for pentylenetetrazol (PTZ) kindling after TBI in which daily 35 mg/kg PTZ challenge occurs 1 or 7 days after TBI (top) or concurrent M084 (10 mg/kg) 1 h before PTZ challenge (bottom). End-point criteria for kindling of mice was behavioral evidence of tonic–clonic motor seizure exhibited through tonic hindlimb extension. **(B)** Representative recordings of surface cortex and hippocampal depth electrode EEGs during the three distinct stages of PTZ-induced motor seizure activity: myoclonic jerks, forelimb clonus, and tonic hindlimb extension. **(C)** Rate of PTZ kindling initiated in sham mice, mice 1 day after TBI, and mice 7 days after TBI. Bars summarize the mean number of days required to elicit the motor behavioral activity. *p < 0.05 vs. sham. *n* = 6–8 mice per group. **(D)** Cumulative progression of mice achieving clonic seizures after daily PTZ challenge, as a percentage of total animals per group. TBI mice challenged after delaying 1 and 7 days showed similar rates of kindling and exhibited accelerated seizure activity compared to sham mice. **(E)** Rate of PTZ kindling 7 days after sham or TBI compared to mice treated with M084 from paradigm shown at the bottom figure of Panel **(A)**. Bars summarize the mean number of days required to elicit the motor behavioral activity. M084 treatment significantly impeded the progression of seizures compared to untreated TBI animals. **(F)** Cumulative progression of M084-treated mice achieving clonic seizures after daily PTZ challenge, as a percentage of total animals per group. *p < 0.05 vs. sham + veh., ^†^*p* < 0.05 vs. TBI + veh. *n* = 6–7 mice per group. Statistical comparisons were made with the Mann–Whitney *U* test. All data bars represent the mean ± SEM.

We previously reported that in our model of CCI-TBI to adult mice, approximately one-third of animals exhibited epileptiform seizure activity within 6 days following injury, as measured by cortical EEG electrodes (Vigil et al., 2019). The variables causing only a fraction of TBI events leading to epileptic seizures is a yet unanswered phenomenon in both preclinical and clinical studies. Therefore, we investigated seizure susceptibility within the CCI-TBI model to gain a greater understanding as to the etiology of hyperexcitability spread occurring postinjury. CCI-TBI accelerates chemical kindling epileptogenesis in rats, induced by the GABA_*A*_ receptor antagonist PTZ (Eslami et al., 2016; Huttunen et al., 2018). In addition, CCI-TBI results in greater seizure susceptibility to single-dose subconvulsant PTZ (Bolkvadze et al., 2016; Lu et al., 2020). In our seizure-susceptibility paradigm, 1 or 7 days after the experimental procedure, mice were challenged with a subconvulsant dose of PTZ (35 mg/kg s.c.) once daily to elicit chemical kindling epileptogenesis (Figure 7A). The progression of motor seizure behavior was observed and timed by direct observation linked to EEG activity (Figure 7B). During the PTZ kindling that was initiated 1 day post-TBI (Figure 7C), the average latencies to motor seizures in mice were myoclonic jerks, (sham) 9.5 ± 0.6 vs. (TBI) 4.4 ± 0.6 days; forelimb clonus, (sham) 11.3 ± 0.7 vs. (TBI) 5.6 ± 0.7 days; and tonic–clonic seizure, (sham) 12.5 ± 0.8 vs. (TBI) 7.9 ± 0.7 days (*p* < 0.001, Mann–Whitney *U* test, *n* = 6–8 per group). In the TBI group, five out of eight mice experienced lethal seizures on the second occurrence of tonic–clonic epileptic activity. It is important to note that these PTZ-induced seizures were induced in the reported presence of early pathophysiological changes in the brain such as inflammation and microgliosis 1–3 days after injury (Vigil et al., 2019). In mice with PTZ challenge initiated 7 days after TBI, the average latencies to seizure were myoclonic jerks, (sham) 9.8 ± 0.4 vs. (TBI) 3.6 ± 0.7 days; forelimb clonus, (sham) 12.0 ± 0.6 vs. (TBI) 4.3 ± 0.8 days; and tonic–clonic seizure, (sham) 13.1 ± 0.6 vs. (TBI) 5.6 ± 1.2 days (Figure 7C, *p* < 0.01, Mann–Whitney *U* test, *n* = 6–7 per group). The progression of clonic activity was significantly escalated in the kindling mice initiated 1 and 7 days after TBI (Figure 7D). Thus, we demonstrate that TBI mice exhibit greater PTZ-induced seizure susceptibility overall as compared to sham control mice.

We wished to understand if the TRPC channel upregulation after TBI could be targeted to impede epileptogenic activity. Since we observed multiregion alterations in TRPC channel expression after TBI, we tested post-TBI seizure susceptibility in the presence of TRPC4/TRPC5 channel blockade. We tested a group of sham and TBI animals in which 7 days after surgery, M084 (10 mg/kg, s.c.) was given 1 h before each PTZ kindling challenge (35 mg/kg) (Figure 7E). Therefore, at the time of PTZ challenge, brain concentrations of M084 are likely within the micromolar range (Yang et al., 2015). Seizure susceptibility was standardized according to the latency to forelimb clonus lasting >5 s. We observed that TRPC4/TRPC5 antagonist impeded the rate of epileptogenesis (Figure 7E). Within sham mice, M084 significantly prolonged the number of PTZ challenges required to elicit clonic (*p* = 0.0232) and tonic–clonic (*p* = 0.0128) seizures compared to sham mice given vehicle (Mann–Whitney *U* test, *n* = 5–8 mice per group). The number of PTZ challenges required to reach myoclonic jerks (Supplementary Figure 5A), forelimb clonus (Supplementary Figure 5B), and tonic hindlimb extension (Supplementary Figure 5C) seizure behaviors were also significantly prolonged in the TBI group pretreated with M084 compared to vehicle-treated TBI mice. M084 administration resulted in TBI mice requiring an average of 7.3 ± 0.6 days to demonstrate myoclonic jerks, 10.6 ± 0.5 days for clonic seizures, and 11.0 ± 0.6 days to reach tonic–clonic seizure behavior. Thus, M084 treatment delayed the progression of seizures, displaying a greater latency than CCI-TBI mice without treatment and similar to the rate of progression experienced by sham animals (Figures 7E,F). As explored by our epileptogenic progression model, these results suggest that TRPC4/TRPC5 channels strongly contribute to seizure susceptibility after TBI. Previous TRPC knockout models ascertained susceptibility in response to pilocarpine, which directly stimulate mAChRs (Phelan et al., 2012). Our novel approach determined seizure susceptibility in corroboration of acquired epileptogenesis after TBI, and these findings are consistent with a TRPC-specific role in contribution to neuronal hyperexcitability.

Finally, we tested the effect of M084 drug treatment on TBI prior to an excitotoxic challenge with PTZ (Supplementary Figure 5D). Thirty minutes after recovery from sham or TBI, mice were delivered vehicle or M084 (10 mg/kg). Seven days after the procedure, mice were injected with a convulsant dose of PTZ (60 mg/kg) in order to test drug-modulating effects at the time of TBI. Latency to seizure behavior was recorded as a measure of seizure susceptibility. Sham animals given M084 did not exhibit significantly altered latency to the three stages of PTZ-induced seizure compared to shams given vehicle. In TBI mice, M084 only marginally prolonged clonic seizure latency, and this was not statistically significant in comparison to vehicle-injected TBI mice (*p* = 0.211). However, all of the TBI mice treated with M084 were protected from tonic–clonic activity, whereas four out of five TBI mice given vehicle had tonic seizures. Therefore, M084 treatment at the time of initial trauma may be efficacious in modulating post-TBI seizure susceptibility and excitoxicity.

## Discussion

In this study, we investigate the mechanism of hyperexcitatory activity in the cortex and hippocampus that contributes to posttraumatic seizure susceptibility in a mouse model of TBI-induced epileptogenesis. TRPC4/TRPC5-containing channels were significantly upregulated in a cell-specific manner in the hippocampus and cortex after CCI brain injury (Tables 1, 2 and Figure 2). Using the cFos/TRAP mouse model, we determined the fraction of active neurons during cortical impact and 7 days after TBI. Dynamic neuronal activity could be clearly visualized and assessed post-TBI (Figure 3 and Supplementary Figure 3), and we found that TRPC4/TRPC5 blockade attenuated trauma-directed excitability. Furthermore, we determined that in response to trauma, DGGCs demonstrate greater cholinergic sensitivity via mAChR-mediated increases in intracellular [Ca^2+^] (Figure 4). TRPC4/TRPC5 channels could be directly, pharmacologically targeted to either enhance or reduce neuronal activity (Figure 5). AChE activity was significantly decreased in the cortex and DG (Figure 6). In conjunction with previous reports of parallel increases in M_1_R expression and modulation (Jiang et al., 1994; Delahunty et al., 1995a, b) and trauma-induced changes to ACh trafficking (Dewar and Graham, 1996; Ciallella et al., 1998; Shao et al., 1999), our findings showed that TBI has the potential to facilitate cholinergic overdrive by a multipronged, maladaptive compensation. We posit that this cholinergic hyperactivation may contribute to posttraumatic hyperexcitation of neurons. Through both functional and behavioral studies, we demonstrate that selective pharmacological block of TRPC4/TRPC5 channels *in vivo* limits trauma-induced hyperexcitatory activity in the cortex and hippocampus (Figure 3) and impedes TBI-directed epileptiform seizure susceptibility (Figure 7).

### Visualization of Trauma-Induced Neuronal Excitability

Previous studies show significant but transient increases in cortical and hippocampal cFos+ expression 2–3 h after CCI-TBI and that cFos activity diminishes at 1–3 days after injury (Yang et al., 1994; Czigner et al., 2004; Villasana et al., 2014, 2019). Using cFos-TRAP transgenic mice, we were able to detect cFos activity that occurred over a continuous time range. This provided more than a snapshot that only occurs immediately prior to the time of sacrifice. A limitation of this model, as used for histology, is that we are unable to pinpoint the precise timing of cFos activation at the level of single neurons. However, the 4-OHT activation window provides prolonged time of “recording” as a greater opportunity for spatial and quantitative assessment of ensemble neuronal activity. We also are mindful that indirect cFos activity can occur at the same time that may not be related to TBI. Total time for “trapping” of cFos+ neurons, that is, Cre recombinase during cFos activity, is dictated by the pharmacokinetics of 4-OHT, in which 4.5 mg/kg is the minimum effective concentration (Valny et al., 2016). A lingering concern is that 4-OHT administration itself may induce off-target effects (Wust et al., 2020). We addressed these concerns by testing the TBI mice alongside sham controls that were handled similarly, in which we report background/basal activity with 4-OHT to be nominally small. We demonstrate evaluation of cFos-TRAP activity as a novel and improved representation of the ongoing progression of hyperexcitability in the days after the initial trauma.

### Cholinergic Mechanisms of Posttraumatic Adaptation and Plasticity

The compensatory transformation of key neuronal pathways into primed, hyperexcitatory networks involves destabilization of the balance of excitation and inhibition (Avramescu and Timofeev, 2008; Ding et al., 2011; Cantu et al., 2015; Boychuk et al., 2016; Ibrahim et al., 2016; Neuberger et al., 2019). As a signaling neurotransmitter responsible for modulating spike activity in the hippocampus, ACh differentially affects synaptic plasticity through both excitation and inhibition and in a cell-type-specific manner (Lawrence et al., 2006; Dannenberg et al., 2015, 2017). The translational role of cholinergic signals associated with TBI has centered on mechanistic and clinical attempts to leverage AChE inhibition to curtail cognitive deficits and facilitate recovery (Carrillo-Mora et al., 2017) but without significant success. Recent data suggest that pretraumatic cholinergic deficit may underlie the severity of posttraumatic cognitive deficits (Cherian et al., 2019). This emerging perspective would be highly intriguing to explore in seizure susceptibility phenotypes. Despite chronic posttraumatic deficits in cholinergic hypofunction, initial acute responses to TBI result in large increases of acetylcholine release (Shin and Dixon, 2015). Our findings point toward a concerted role of cholinergic changes in response to injury, and we report that maladaptive responses mediated by TRPC channels may provoke greater posttraumatic excitotoxicity. We found that AChE is decreased non-transiently after CCI-TBI (Figure 6) and may in fact contribute to abnormal hyperexcitability following TBI.

Stimulation of nAChRs and mAChRs increases long-term potentiation in the DG (Luo et al., 2008). With the possibility of a widespread increase in cholinergic tone due to a deficit in AChE activity, nAChR-mediated actions should also be considered. We previously reported only a limited effect of nAChR block on DGGC excitability (Carver and Shapiro, 2019), and the addition of hexamethonium to brain slice studies here did not significantly change neuronal activity in response to TBI. However, others have reported posttraumatic deficits in nAChR expression and decreased binding after CCI-TBI (Verbois et al., 2002; Hoffmeister et al., 2011). Pharmacological targeting of nAChR may reduce cognitive deficits following TBI (Verbois et al., 2003); however, α7 nAChR knockout mice do not exhibit differences in posttraumatic tissue loss or neuroinflammation (Kelso et al., 2006). Some of these findings could be obfuscated by compensatory increase in synaptic ACh activity (e.g., via less AChE-mediated catalysis) not detected by the nAChR radioligand binding assays used in those studies. Most commonly, a standard approach has been to assess the mechanisms of mAChR and nAChR signaling independently of one another (Kelso and Oestreich, 2012), but we suppose there is as yet unexplored cross-talk between these cholinergic systems that requires further investigation.

Our study here is the first to characterize a trauma-induced alteration of TRPC channel activity, via both rapid GPCR-mediated signaling pathways, and more long-term alterations in gene transcription, which each promote hyperexcitability at both the site of trauma and in the hippocampus, which is not proximal to the site of insult but synaptically connected via cholinergic transmission. Whereas pilocarpine-induced seizure models circumvent physiological network circuits in evoking widespread mAChR-mediated hyperexcitation in the hippocampus, our TBI studies demonstrate that, in practice, selective neuronal hyperactivation occurs. We find that DGGCs are most affected, but CA3 and CA1 pyramidal neurons remained relatively quiescent (Figure 3). Based on the TRAP neuronal activity assay, we surmise that networks in the DG effectively gate the trisynaptic hippocampal circuit against propagating hyperexcitatory signals in our model of TBI. Although the CA3 region demonstrated reduced AChE activity, the posttraumatic neuronal activity of CA3 pyramidal neurons was not significantly altered. We postulate that in CA3 and CA1, this is due to more pronounced inhibitory interneuron activity (Yi et al., 2021) that compensates for the reduction in AChE. Evidence for this supposition includes that G_*q/*__11_-coupled receptors, including M_1_Rs, prime long-term potentiation at GABAergic synapses (Morales-Weil et al., 2020), thus amplifying regulation of glutamatergic principal cells by cholinergic pathways. The scope of this present study was to illuminate the functional response to TBI by excitatory, principal neurons, but the investigation of the role of interneuron cholinergic signals in the regulation of TRPC channel activity in GABAergic neurons is needed to fully understand the overall pathophysiological response to TBI. ACh also activates astrocytic excitation of hilar inhibitory neurons that promote GABAergic inhibition of local circuits (Pabst et al., 2016). The expression and role of TRPC channels in astroglial activation, ion channel homeostasis, and store-operated Ca^2+^ entry is likely affected by cholinergic disruptions (Verkhratsky et al., 2014), but has not been investigated in the context of TBI.

### TRPC Channel Subunit Composition in TBI Pathophysiology

It is intriguing that the DG, CA3, and parietal cortex exhibited parallel changes to TRPC4 expression after TBI, but changes in TRPC5 expression were limited. The observed asymmetrical changes in TRPC4 and TRPC5 after TBI requires further investigation and focus on cell-type specificity. The heteromerization of TRPC channels remains a complex and unresolved area of investigation (Kim et al., 2019). TRPC5 has an established role in the development and neurite outgrowth with some localization to presynaptic terminals (Greka et al., 2003), which may indicate a greater role of TRPC5 in the maintenance of homeostasis. This may also be the case for TRPC1, which had in this study displayed a significantly lower shift in transcriptional expression after TBI, compared to TRPC4 and TRPC5. Our results show that TRPC4 and TRPC5 are upregulated in parallel in the cortex, in which we found substantial neuronal hyperactivity occurring 7 days after TBI, but TRPC4 transcription was preferentially upregulated in the hippocampus. Despite the demonstration that TRPC5 plays a partial role in epileptogenic excitability (Tai et al., 2011; Zheng and Phelan, 2014), it is possible that TRPC4 and TRPC5 channels cannot be separated apart as therapeutic targets for preventing seizure activity; however, recent advancements in structure-activity studies may challenge such questions that we cannot yet answer (Wang et al., 2020). Furthermore, competition may exist in the preferential development and trafficking of TRPC channel subunits to the cell membrane, dependent on the type of neuron and its state, whether quiescent, excited, hyperexcited, low or high intracellular [Ca^2+^], etc. Further investigations of TRPC1/4/5 channel permutations *in vivo* are needed in order to determine properties that are related to cation permeability and selectivity, particularly Ca^2+^ and modulation of gating by intracellular second messengers (Storch et al., 2012).

We have not directly addressed the role of TRPC3 or TRPC6 in hippocampal/cortical functions in this study. TRPC channels containing both subunits are present in hippocampal neurons, with TRPC6 prominently expressed in the DG (Kim et al., 2013). TRPC3 and TRPC6 have been suggested to play contrasting roles in excitoxicity in a seizure induction model of epilepsy, with TRPC3 upregulated and TRPC6 downregulated in the hippocampus after pilocarpine-induced seizure (Kim et al., 2013). There is evidence that TRPC6 may partly contribute to neuronal death in the DG in pilocarpine-induced status epilepticus (Kim and Kang, 2015). However, we have previously demonstrated that selective blockade of TRPC4/TRPC5 channels largely reduces the changes in intracellular [Ca^2+^] in DGGCs after M_1_R stimulation (Carver and Shapiro, 2019). Moreover, in pilocarpine-induced epilepsy, TRPC4/TRPC5 blockade significantly inhibits seizure severity. Thus, given that >70% of the mAChR-induced cation influx in DGGCs is due to TRPC4/TRPC5-containing channels, we hypothesize that pathophysiological modulation of TRPC4/TRPC5 after TBI represents an overarching regulatory control of hyperexcitability. Having demonstrated the robust upregulation of TRPC4 expression after TBI, the efficacy of TRPC4/TRPC5 blockade to impede the process of epileptogenesis in our PTZ kindling model supports our hypothesis (Figure 7E). TRPC4/TRPC5 block with M084 was moderately effective at impeding kindling in sham control mice, slowing the clonic and tonic–clonic phases of motor seizures by approximately four challenges of subconvulsant PTZ. However, M084 attenuated the TBI-induced hyperexcitability in returning the number of subconvulsant challenges required similar to that of sham baseline conditions (Figures 7E,F). M084 did not demonstrate overt protective effects against excitotoxic challenge when given 30 min after TBI (Supplementary Figure 4D). This may suggest that TRPC-targeted utility in dampening seizure susceptibility may be temporally linked to the delayed expressional change of TRPC4-containing channels after TBI. Moreover, the anticonvulsant efficacy of TRPC4/TRPC5 antagonism in the brain will require greater typification beyond chemoconvulsant models (Carver and Shapiro, 2019). Lastly, we have yet to characterize TBI-induced alterations in intracellular signaling pathways that are known to modulate TRPC channels, such as Ca^2+^/CaM, Ca^2+^-induced Ca^2+^ release, PIP_2_ abundance, protein kinases, and interactions with store-operated channels (Singh et al., 2002; Rosker et al., 2004; Tseng et al., 2004; Zhu and Tang, 2004; Ordaz et al., 2005; Xu et al., 2008; Liao et al., 2009; Trebak et al., 2009; Rohacs, 2013; Thakur et al., 2016; Ko et al., 2019; Vinayagam et al., 2020).

### TRPC Channels as a Therapeutic Target for TBI

Intervening to block TRPC4/TRPC5 channels in TBI mice demonstrated a statistically and functionally significant difference in both neuronal activity and seizure response compared to the untreated TBI cohort (Figures 3, 7). TRPC4/TRPC5 antagonism impeded the artificially induced excitability of the PTZ chemical kindling model, which is a highly directed excitatory challenge. Our goal was to identify the early hyperexcitability mechanisms of TRPC channels that may contribute to epileptogenesis but do not preclude other parallel excitatory changes. The efficacy of TRPC4/TRPC5 inhibition in the prevention of spontaneous PTS has yet to be explored and is an important next approach to understanding the mechanistic regulation of TRPC channels after TBI. The inhibition of persistent firing by highly selective TRPC4/TRPC5 antagonists demonstrates the critical role of the channels in hippocampal function (Arboit et al., 2020). In conclusion, the neuromodulatory control of TRPC channels in hyperexcitable brain networks pose interesting potential in the strategic prevention of vulnerability to epileptogenic insults.

## Data Availability Statement

The original contributions presented in the study are included in the article/Supplementary Material, further inquiries can be directed to the corresponding author.

## Ethics Statement

The animal study was reviewed and approved by University of Texas Health San Antonio Institutional Animal Care and Use Committee.

## Author Contributions

CC: study design and writing – original draft. CC, HD, and AS: methodology. CC, HD, AS, and MS: acquisition, analysis, and/or interpretation of data, and writing – reviewing and editing. All authors read and approved the final manuscript.

## Conflict of Interest

The authors declare that the research was conducted in the absence of any commercial or financial relationships that could be construed as a potential conflict of interest.

## Publisher’s Note

All claims expressed in this article are solely those of the authors and do not necessarily represent those of their affiliated organizations, or those of the publisher, the editors and the reviewers. Any product that may be evaluated in this article, or claim that may be made by its manufacturer, is not guaranteed or endorsed by the publisher.

## Supplementary Material

Supplementary Figure 1 TRPC1 protein measurement 7 days after TBI. **(A)** Representative western blots (top) and summarized data (bottom) of TRPC1 protein in CA1 from sham and TBI mice, 7 days after procedure (*n* = 6–7 mice per group). All data bars represent the mean ± SEM. ^∗^*p* < 0.05 vs. sham. TRPC4/5 protein measurement 14 days after TBI. **(B)** Summarized data of western blot protein quantification from mice 14 days after TBI (normalized to the 7 day post-TBI group in Figure 2), in comparison of contralateral and ipsilateral tissue. (*n* = 5 mice per group). All data bars represent the mean ± SEM.

Supplementary Figure 2 Subcellular localization of TRPC4 and M1R in the brain. Shown are mouse brain slices that have been stained with α-TRPC4 (red) and α-M_1_R (green) antibodies and DAPI (blue) for the **(A)** dentate gyrus (DG), **(B)** CA3, **(C)** CA1, and **(D)** parietal cortex layer 5. Hippocampus sub-regions demonstrate co-localization of TRPC4 and M_1_R in principal layer neuron soma, and strong expression of M_1_R in dendrites. Cortex layer 5 neurons show co-localization of TRPC4 and M_1_R in the soma. Scale bars: 100 μm, 20× magnification.

Supplementary Figure 3 The majority of active Layer 5 cortex cells 7 days after TBI are of primarily pyramidal neuron morphology. **(A)** Shown are images of contralateral and ipsilateral layer 5 parietal cortex imaged 7 days after TBI, with tdTomato (red) fluorescence denoting cFos activity. Scale bars: 100 μm. **(B)** Shown is a reconstructed image of complete dentate gyrus coronal slice with tdTomato (red) fluorescence overlayed with DAPI (blue) from a cFos-TRAP mouse 7 days after TBI and activated with 4-OHT.

Supplementary Figure 4 Population Ca^2+^ dynamics in mouse DGGCs 7 days after sham/TBI in response to 0.3 mM ACh. **(A)** Summarized number of DGGCs per brain slice that were responsive in showing at least 1 Ca^2+^ influx event during 0.3 mM ACh. **(B)** Summarized area under the curve (ΔF/F × s) calculations of Ca^2+^ events for control, cTBI, and iTBI cells. **(C)** Histogram population distribution of DGGCs with Ca^2+^ influx according to peak amplitude. **(D)** Histogram population distribution of DGGCs according to Ca^2+^ influx duration. For panels **(C,D)** black bars = control, gray bars = cTBI, striped bars = iTBI. ^∗^*p* < 0.05 vs. control, ^†^*p* < 0.05 vs. cTBI.

Supplementary Figure 5 Time-course of PTZ-induced motor seizure behavior outcomes in sham and TBI mice. Kaplan-Meier plot for mice exhibiting myoclonic jerking behavior **(A)**, forelimb clonus **(B)**, and tonic hindlimb extension **(C)** over time, from data shown in Figure 7E. M084 (10 mg/kg) was delivered 1 h prior to the once-daily dose of subconvulsant PTZ (35 mg/kg). Groups consist of sham (solid line), sham + M084 (dashed line), TBI (red dotted line), and TBI + M084 (dash-dot line). **(D)** Bars represent the latency to the three stages of PTZ-induced motor seizures upon challenge of mice with PTZ (60 mg/kg) 7 days after sham/TBI. Mice were given either vehicle or M084 (10 mg/kg) 30 min after procedure on day 0. ^†^*p* < 0.05 vs. TBI + veh. *n* = 4–6 mice per group. Statistical comparisons were made with the Mann-Whitney *U* test. All data bars represent the mean ± SEM.
