# Supplementary figures and images for: Blockade of TRPC Channels Limits Cholinergic-Driven Hyperexcitability and Seizure Susceptibility After Traumatic Brain Injury
Source: Front Neurosci. 2021 Aug 19;15:681144. doi: 10.3389/fnins.2021.681144 (PMC8416999; doi:10.3389/fnins.2021.681144)

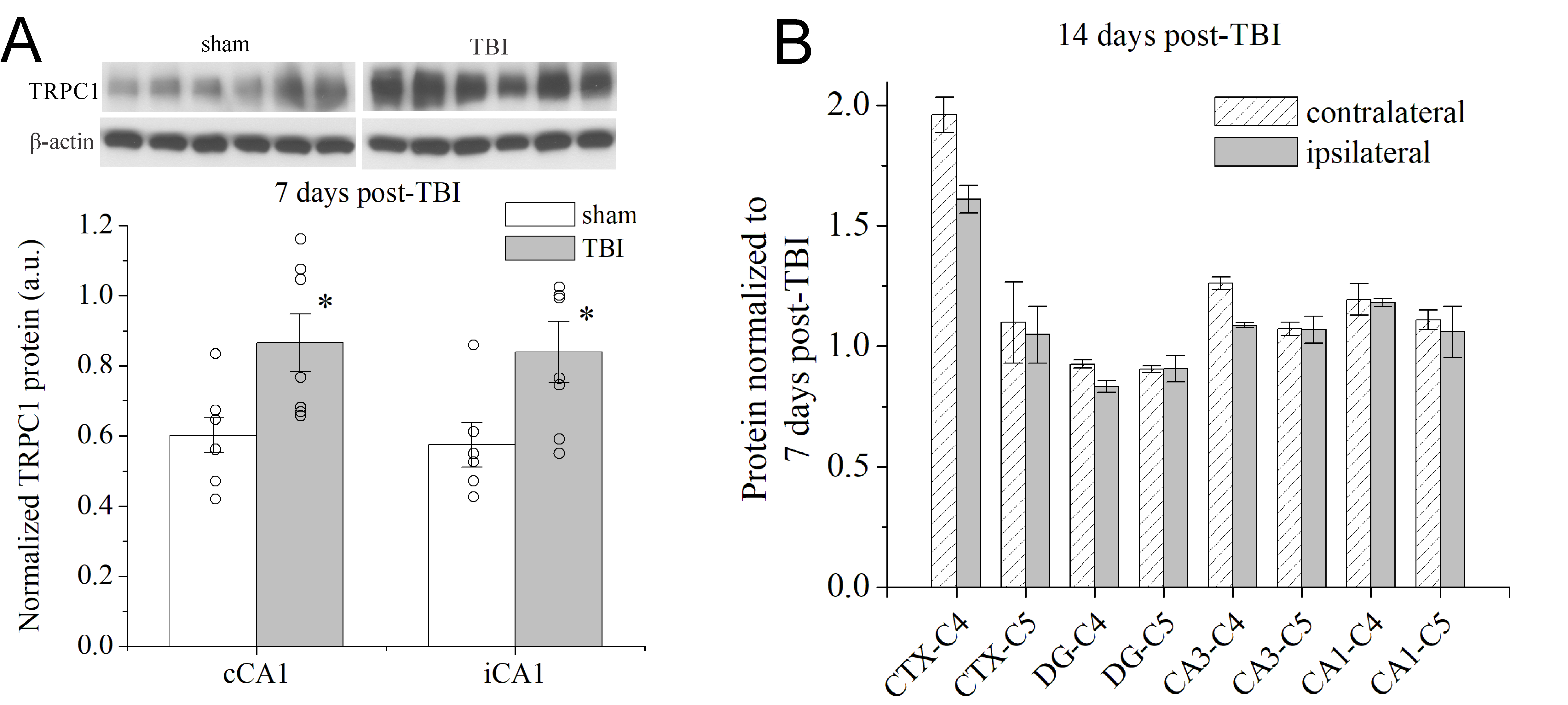

Supplement: Supplementary Figure 1 — TRPC1 protein measurement 7 days after TBI. (A) Representative western blots (top) and summarized data (bottom) of TRPC1 protein in CA1 from sham and TBI mice, 7 days after procedure (n = 6–7 mice per group). All data bars represent the mean ± SEM. ∗p < 0.05 vs. sham. TRPC4/5 protein measurement 14 days after TBI. (B) Summarized data of western blot protein quantification from mice 14 days after TBI (normalized to the 7 day post-TBI group in Figure 2), in comparison of contralateral and ipsilateral tissue. (n = 5 mice per group). All data bars represent the mean ± SEM. [file Image_1.TIF]

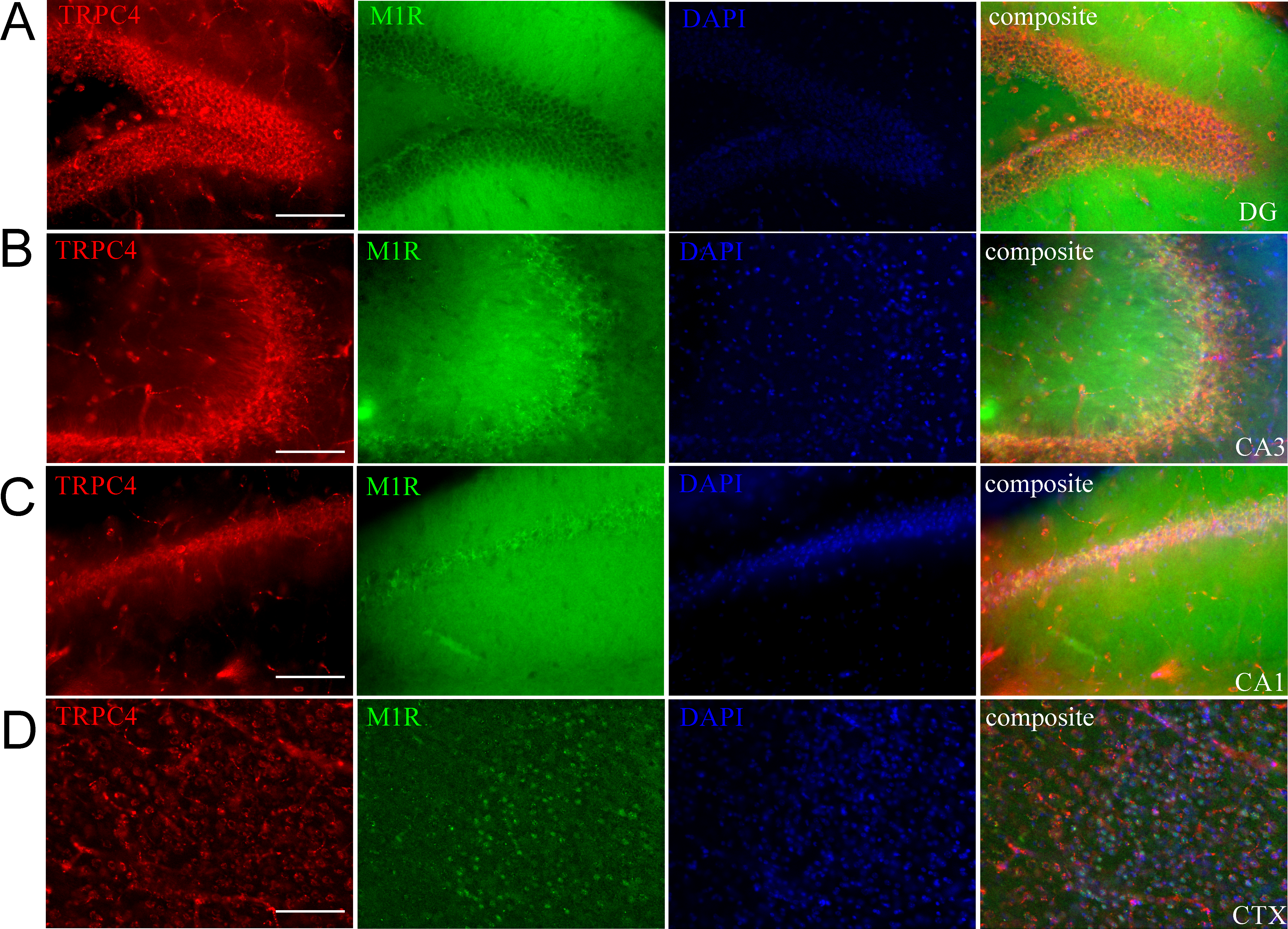

Supplement: Supplementary Figure 2 — Subcellular localization of TRPC4 and M1R in the brain. Shown are mouse brain slices that have been stained with α-TRPC4 (red) and α-M1R (green) antibodies and DAPI (blue) for the (A) dentate gyrus (DG), (B) CA3, (C) CA1, and (D) parietal cortex layer 5. Hippocampus sub-regions demonstrate co-localization of TRPC4 and M1R in principal layer neuron soma, and strong expression of M1R in dendrites. Cortex layer 5 neurons show co-localization of TRPC4 and M1R in the soma. Scale bars: 100 μm, 20× magnification. [file Image_2.TIF]

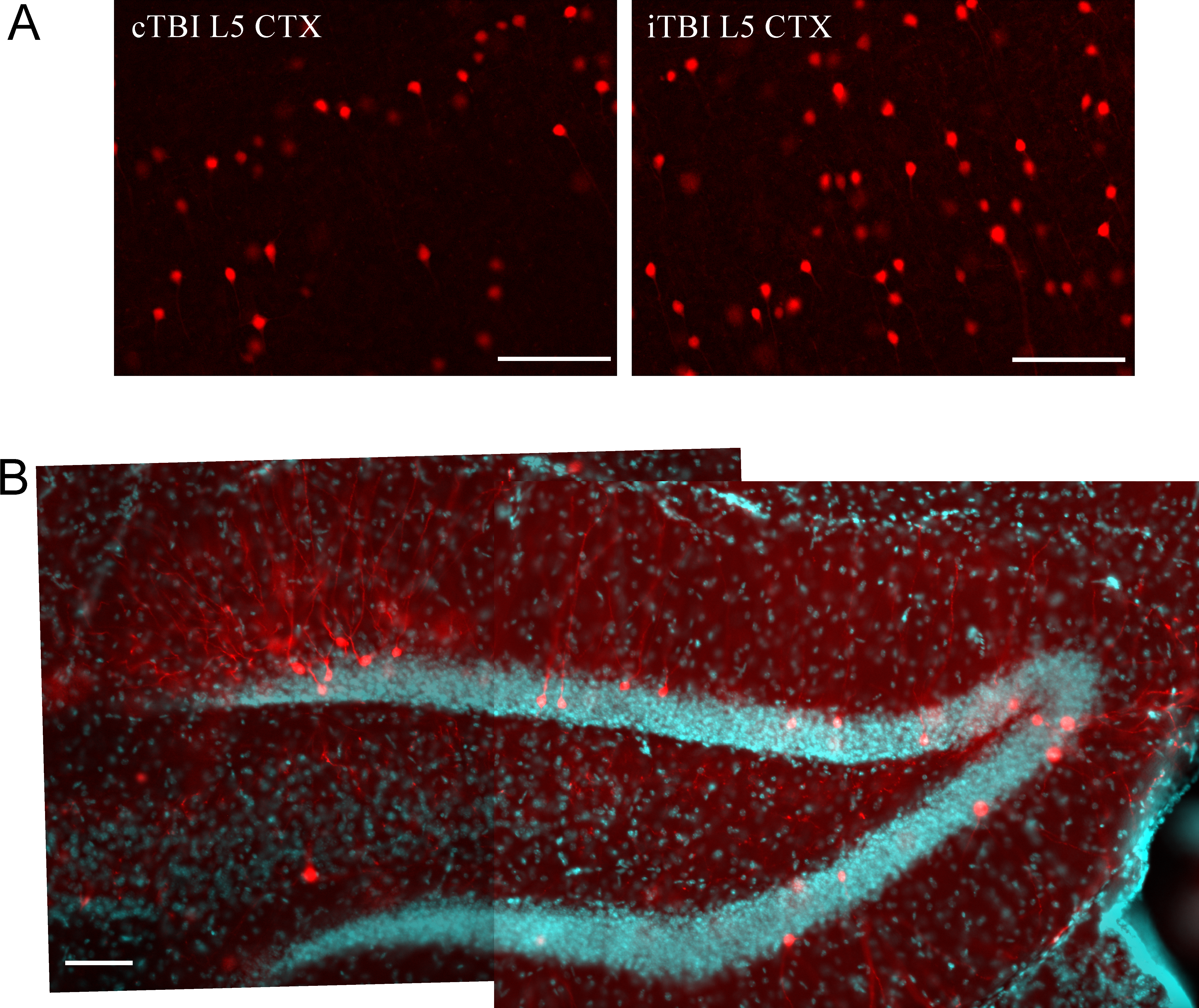

Supplement: Supplementary Figure 3 — The majority of active Layer 5 cortex cells 7 days after TBI are of primarily pyramidal neuron morphology. (A) Shown are images of contralateral and ipsilateral layer 5 parietal cortex imaged 7 days after TBI, with tdTomato (red) fluorescence denoting cFos activity. Scale bars: 100 μm. (B) Shown is a reconstructed image of complete dentate gyrus coronal slice with tdTomato (red) fluorescence overlayed with DAPI (blue) from a cFos-TRAP mouse 7 days after TBI and activated with 4-OHT. [file Image_3.TIF]

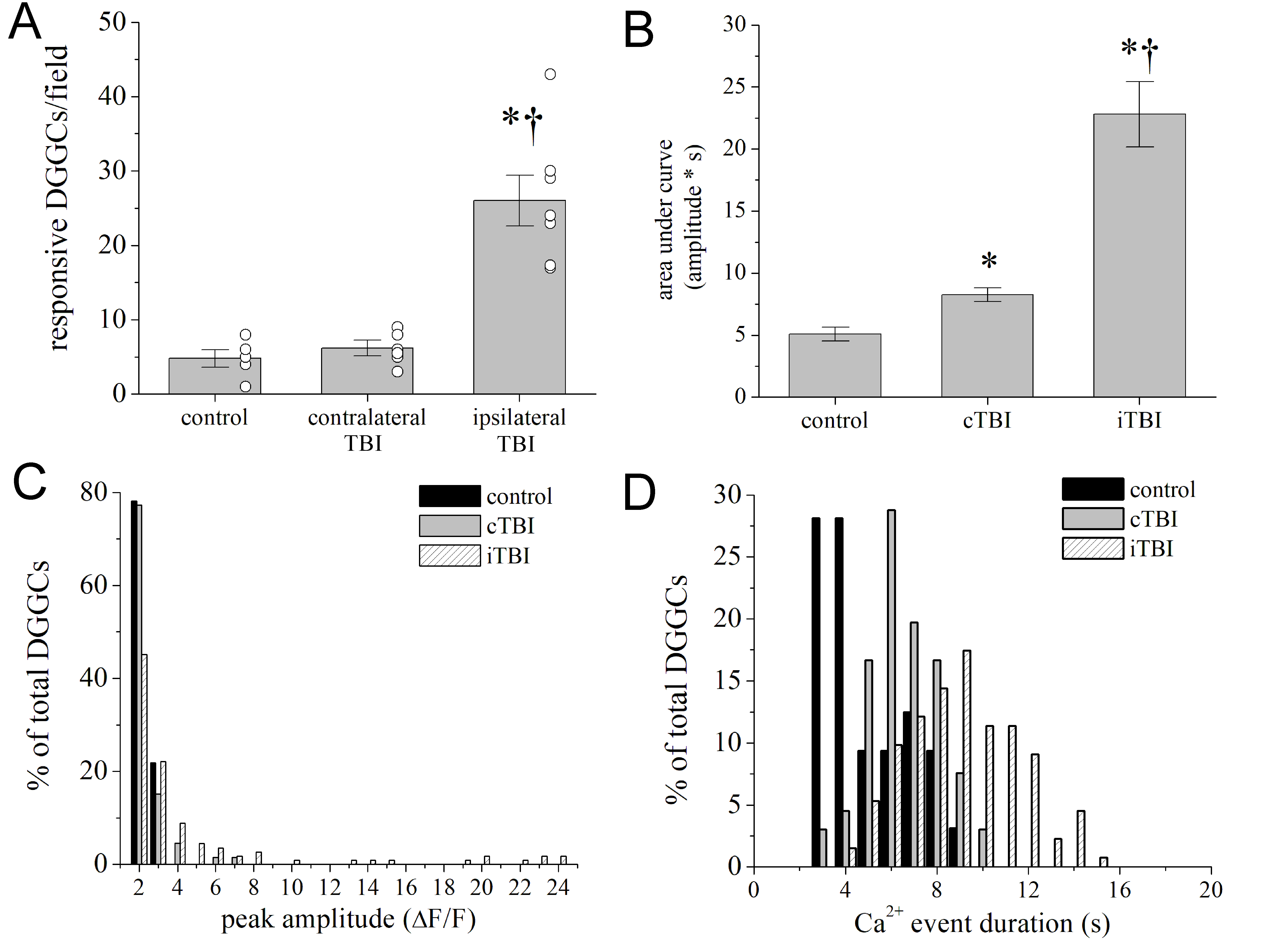

Supplement: Supplementary Figure 4 — Population Ca2+ dynamics in mouse DGGCs 7 days after sham/TBI in response to 0.3 mM ACh. (A) Summarized number of DGGCs per brain slice that were responsive in showing at least 1 Ca2+ influx event during 0.3 mM ACh. (B) Summarized area under the curve (ΔF/F × s) calculations of Ca2+ events for control, cTBI, and iTBI cells. (C) Histogram population distribution of DGGCs with Ca2+ influx according to peak amplitude. (D) Histogram population distribution of DGGCs according to Ca2+ influx duration. For panels (C,D) black bars = control, gray bars = cTBI, striped bars = iTBI. ∗p < 0.05 vs. control, †p < 0.05 vs. cTBI. [file Image_4.TIF]

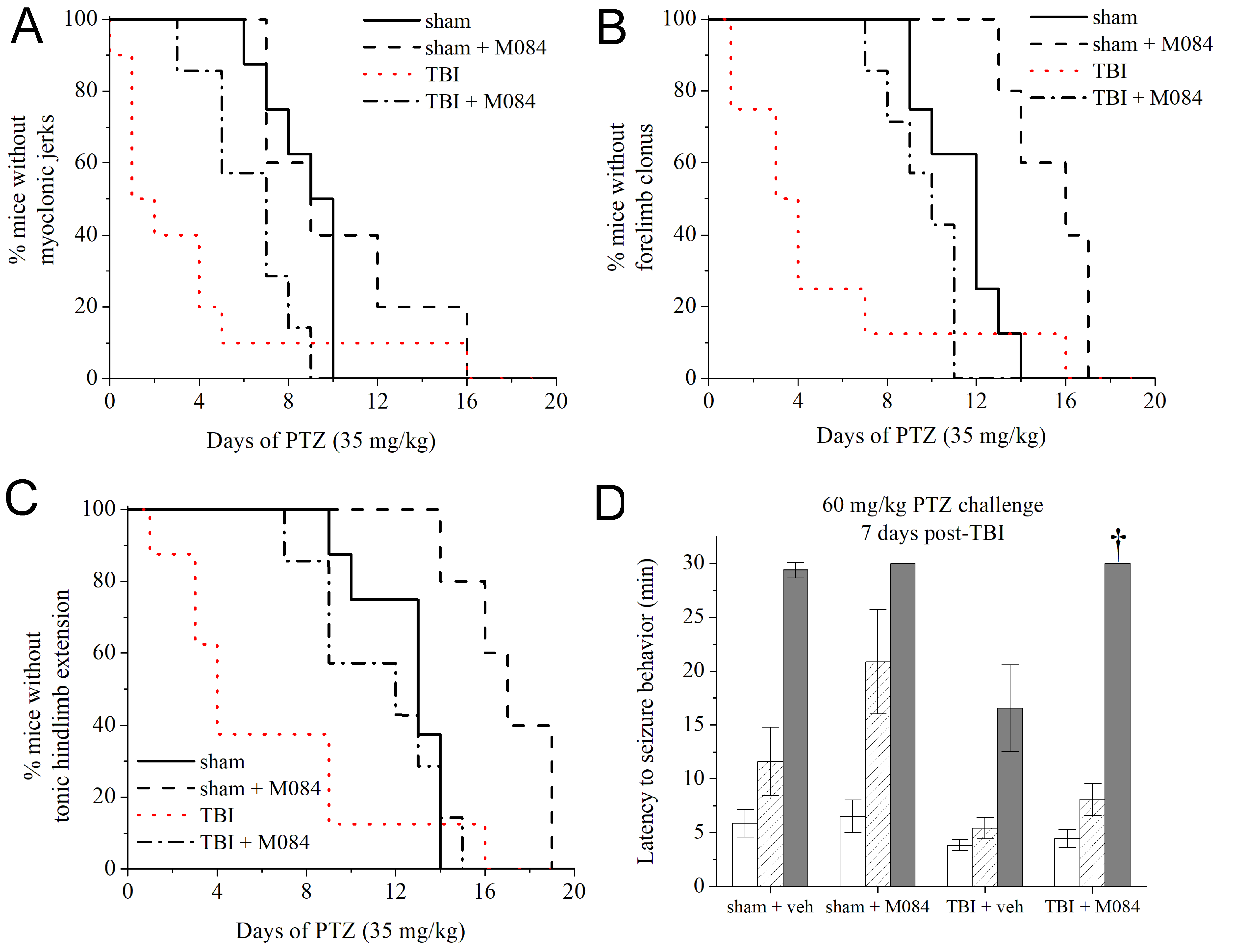

Supplement: Supplementary Figure 5 — Time-course of PTZ-induced motor seizure behavior outcomes in sham and TBI mice. Kaplan-Meier plot for mice exhibiting myoclonic jerking behavior (A), forelimb clonus (B), and tonic hindlimb extension (C) over time, from data shown in Figure 7E. M084 (10 mg/kg) was delivered 1 h prior to the once-daily dose of subconvulsant PTZ (35 mg/kg). Groups consist of sham (solid line), sham + M084 (dashed line), TBI (red dotted line), and TBI + M084 (dash-dot line). (D) Bars represent the latency to the three stages of PTZ-induced motor seizures upon challenge of mice with PTZ (60 mg/kg) 7 days after sham/TBI. Mice were given either vehicle or M084 (10 mg/kg) 30 min after procedure on day 0. †p < 0.05 vs. TBI + veh. n = 4–6 mice per group. Statistical comparisons were made with the Mann-Whitney U test. All data bars represent the mean ± SEM. [file Image_5.TIF]

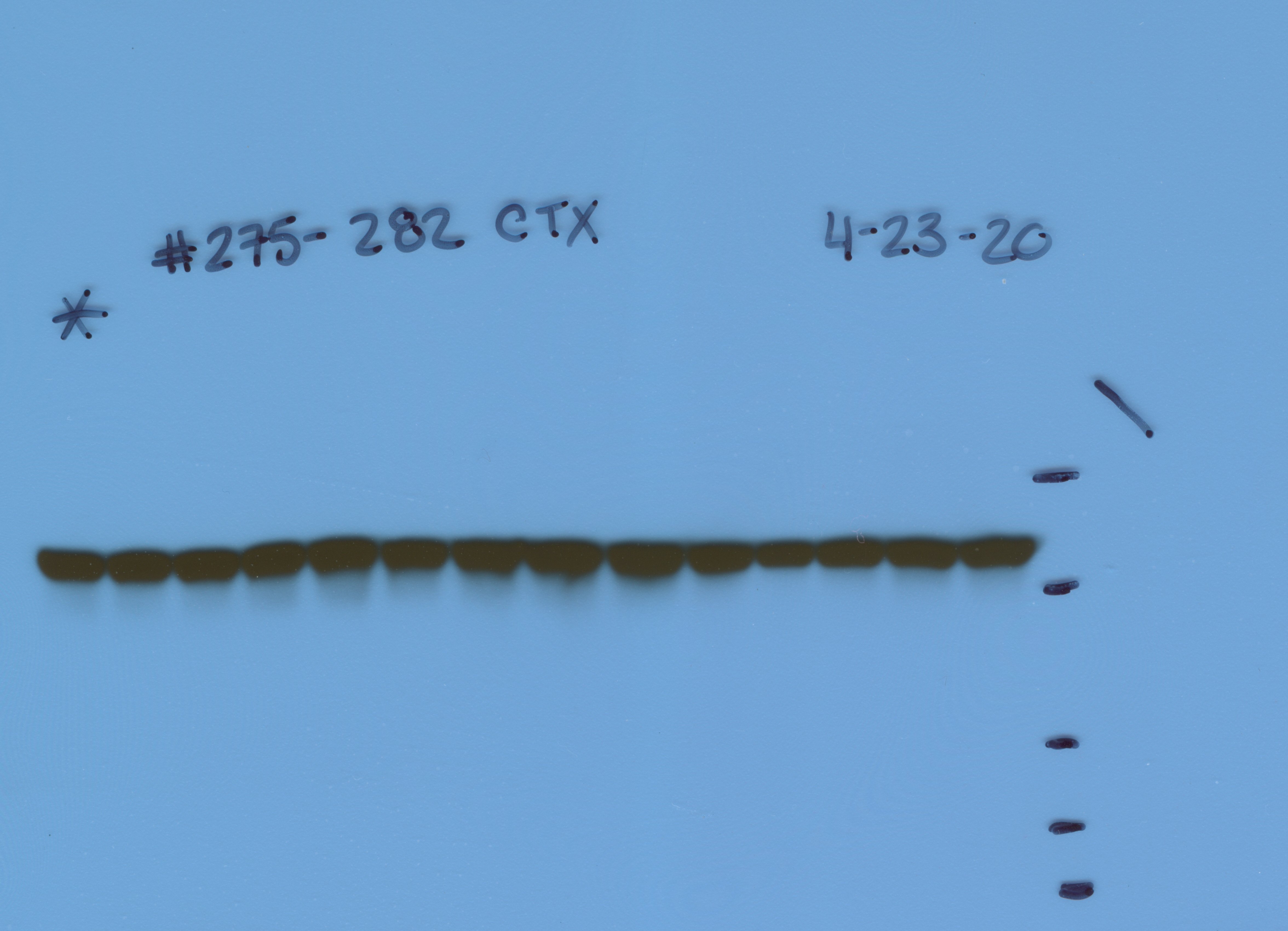

Supplement: Supplementary file 6 [file Data_Sheet_1.ZIP › 2020-4-23 #267-282 CTX Bactin_c4.jpg]

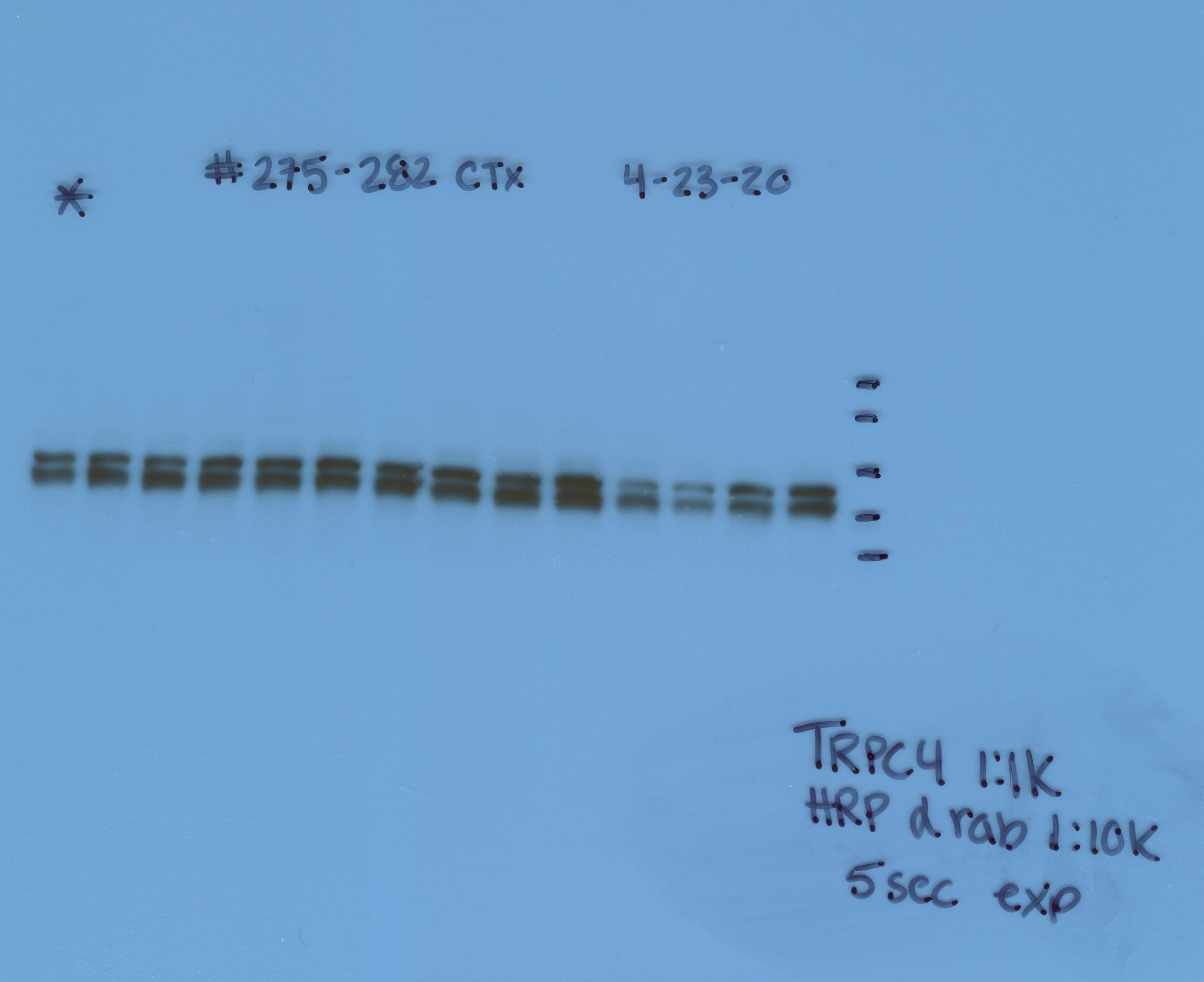

Supplement: Supplementary file 6 [file Data_Sheet_1.ZIP › 2020-4-23 #267-282 CTX TRPC4.jpg]

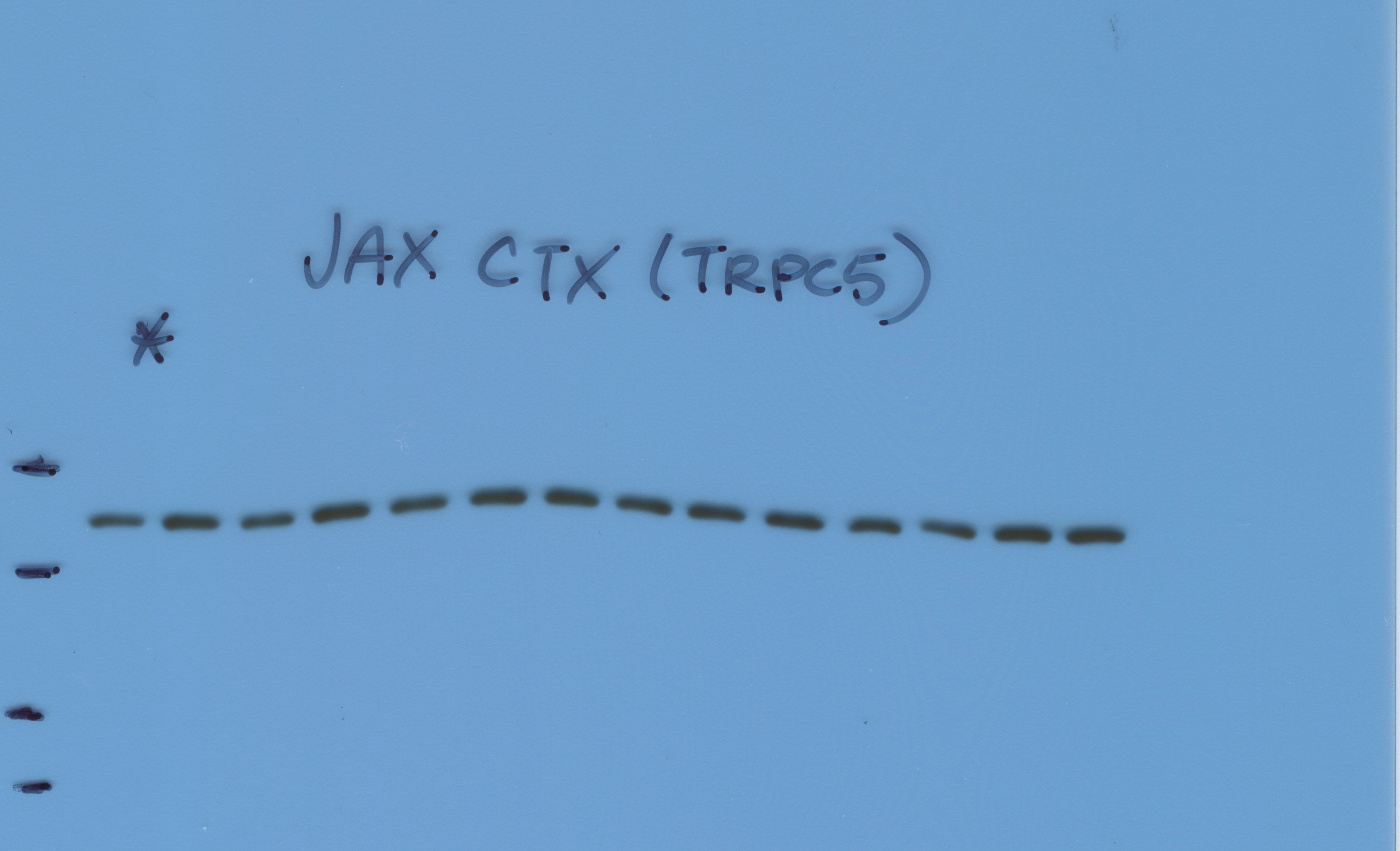

Supplement: Supplementary file 6 [file Data_Sheet_1.ZIP › 2020-5-27 JAX CTX Bactin_c5.jpg]

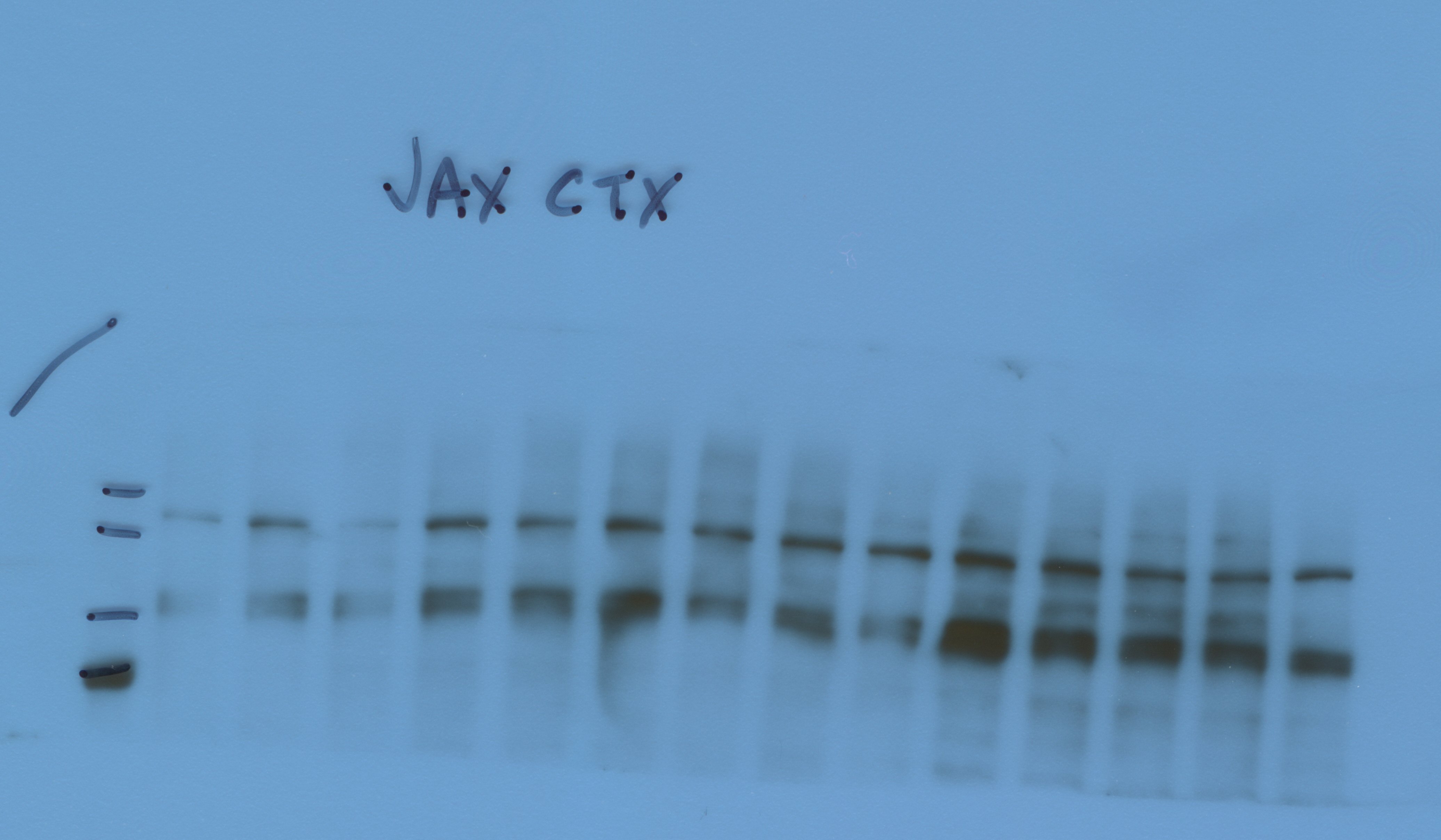

Supplement: Supplementary file 6 [file Data_Sheet_1.ZIP › 2020-5-27 JAX CTX TRPC5.jpg]

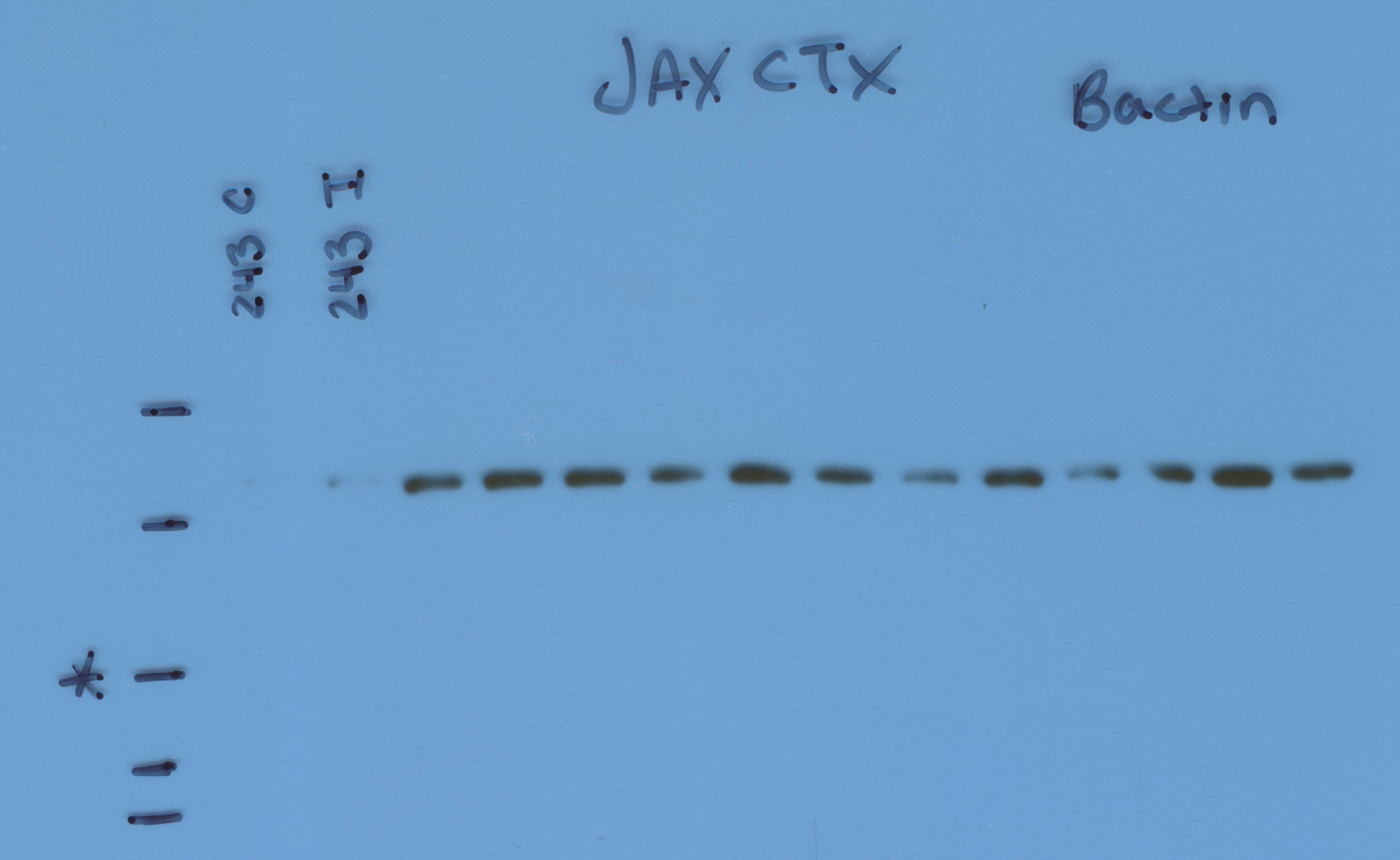

Supplement: Supplementary file 6 [file Data_Sheet_1.ZIP › 2020-6-4 JAX CTX Bactin_c5.jpg]

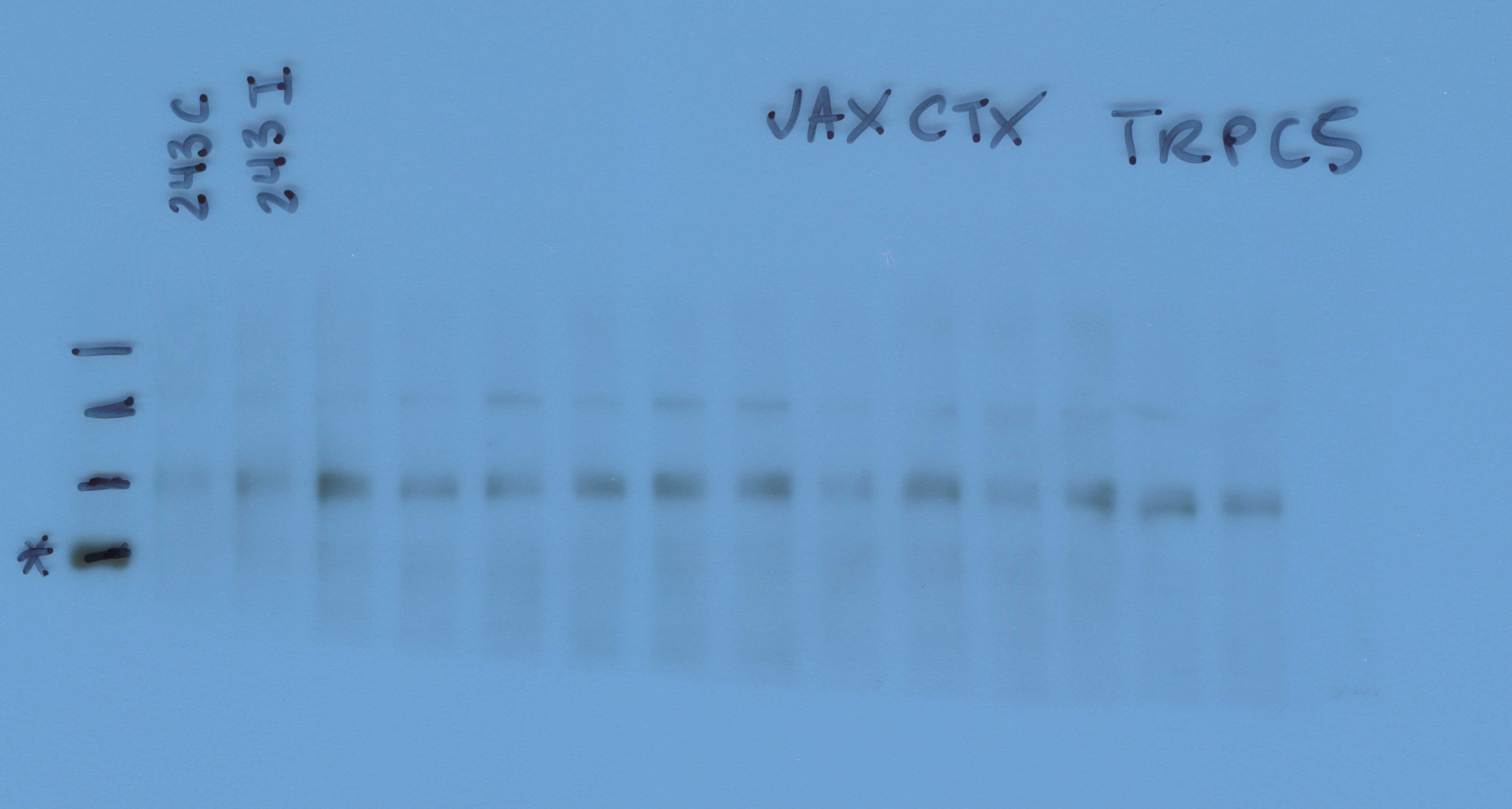

Supplement: Supplementary file 6 [file Data_Sheet_1.ZIP › 2020-6-4 JAX CTX TRPC5.jpg]

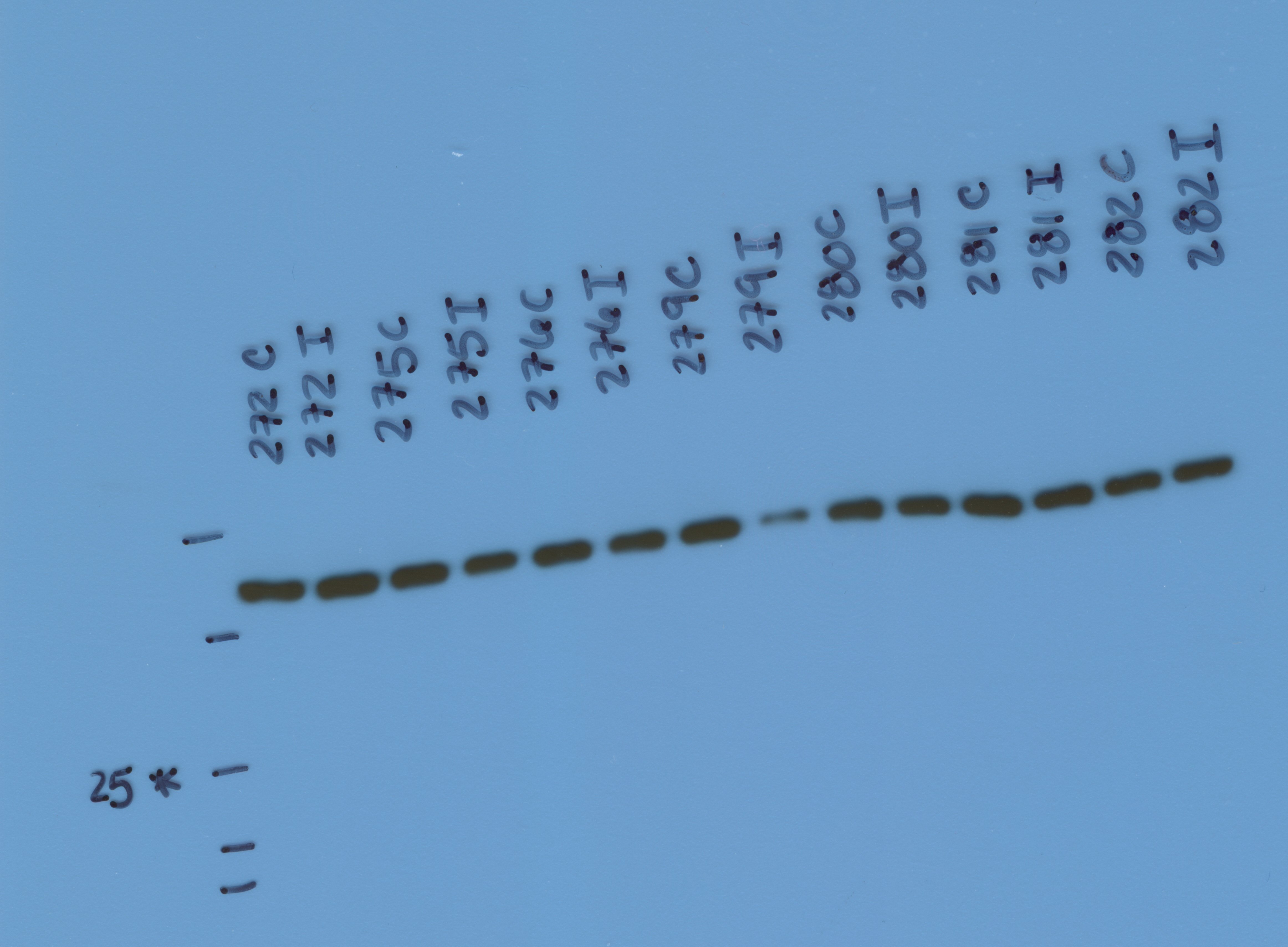

Supplement: Supplementary file 6 [file Data_Sheet_1.ZIP › 2020-6-16 JAX CA1 Bactin_c5.jpg]

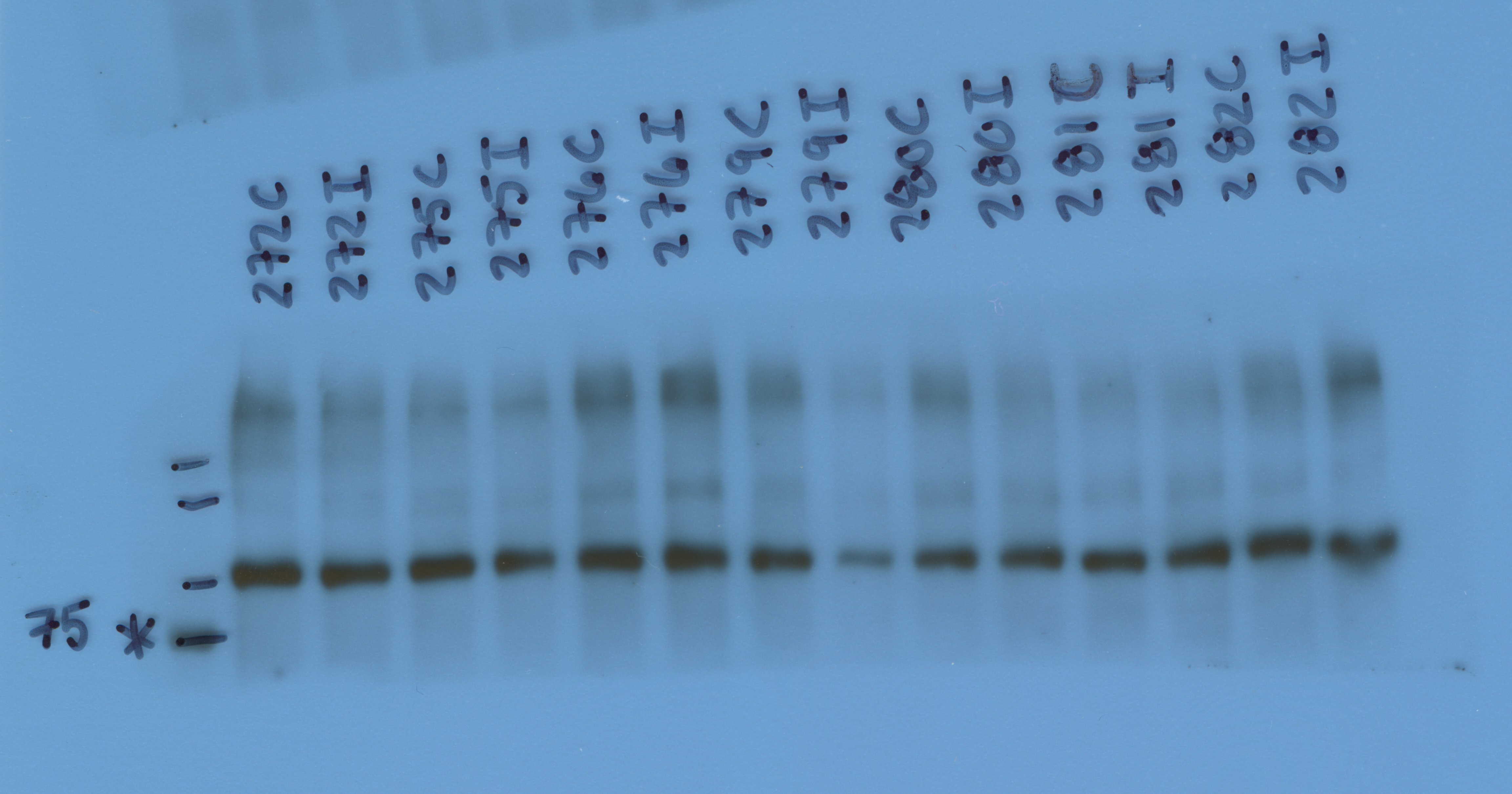

Supplement: Supplementary file 6 [file Data_Sheet_1.ZIP › 2020-6-16 JAX CA1 TRPC5.jpg]

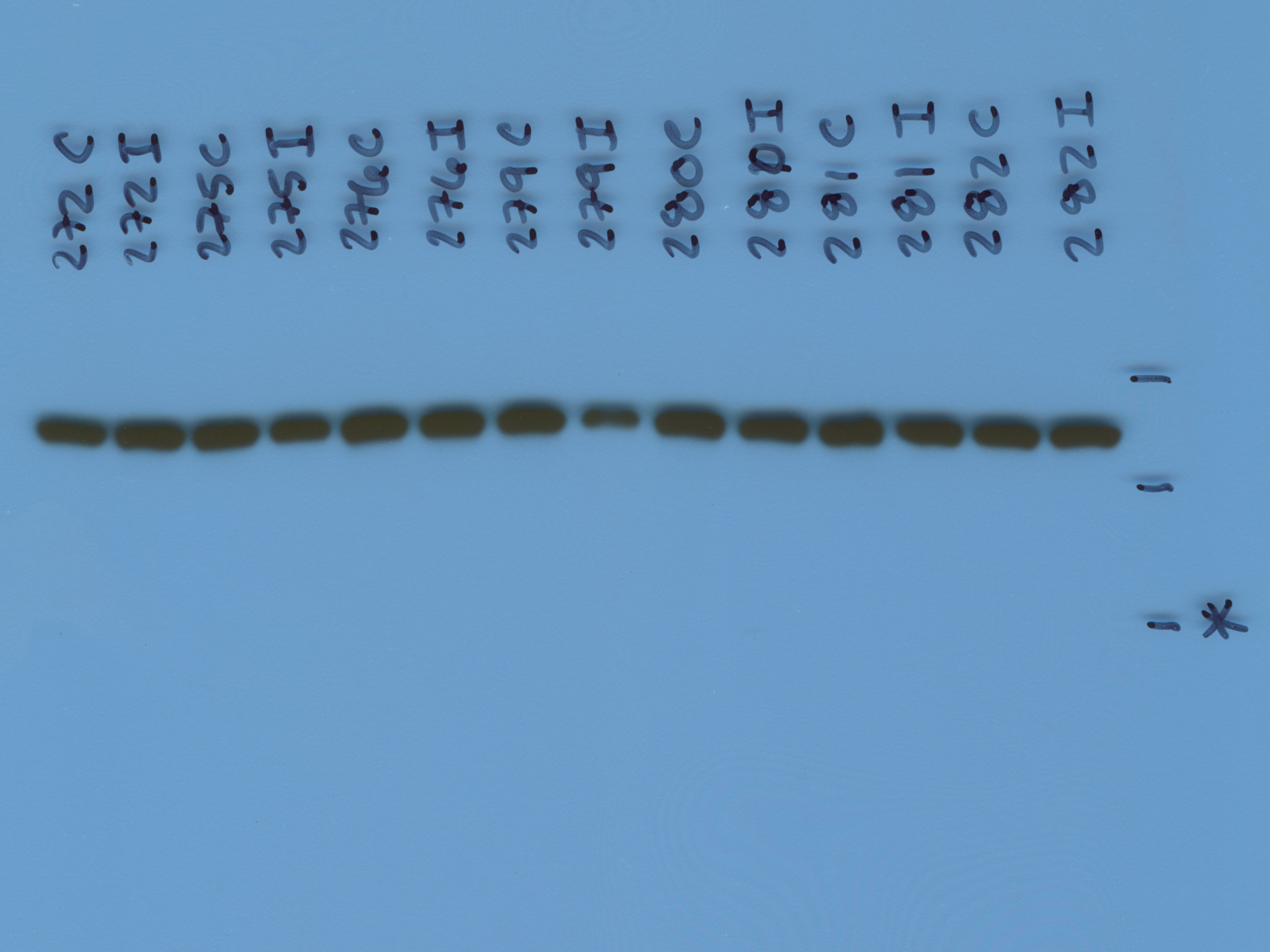

Supplement: Supplementary file 6 [file Data_Sheet_1.ZIP › 2020-6-24 JAX CA1 BACTIN_c1.jpg]

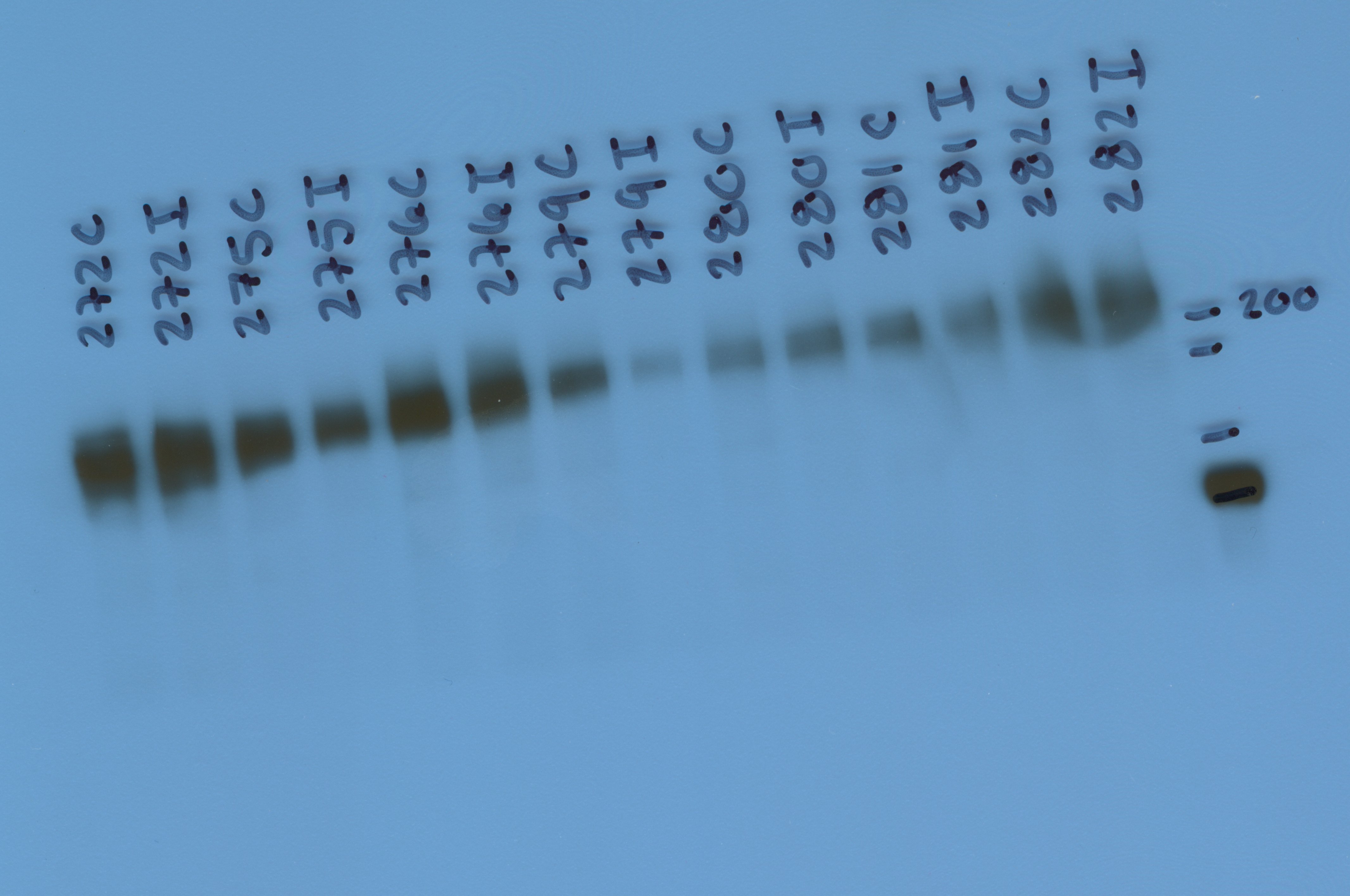

Supplement: Supplementary file 6 [file Data_Sheet_1.ZIP › 2020-6-24 JAX CA1 TRPC1.jpg]

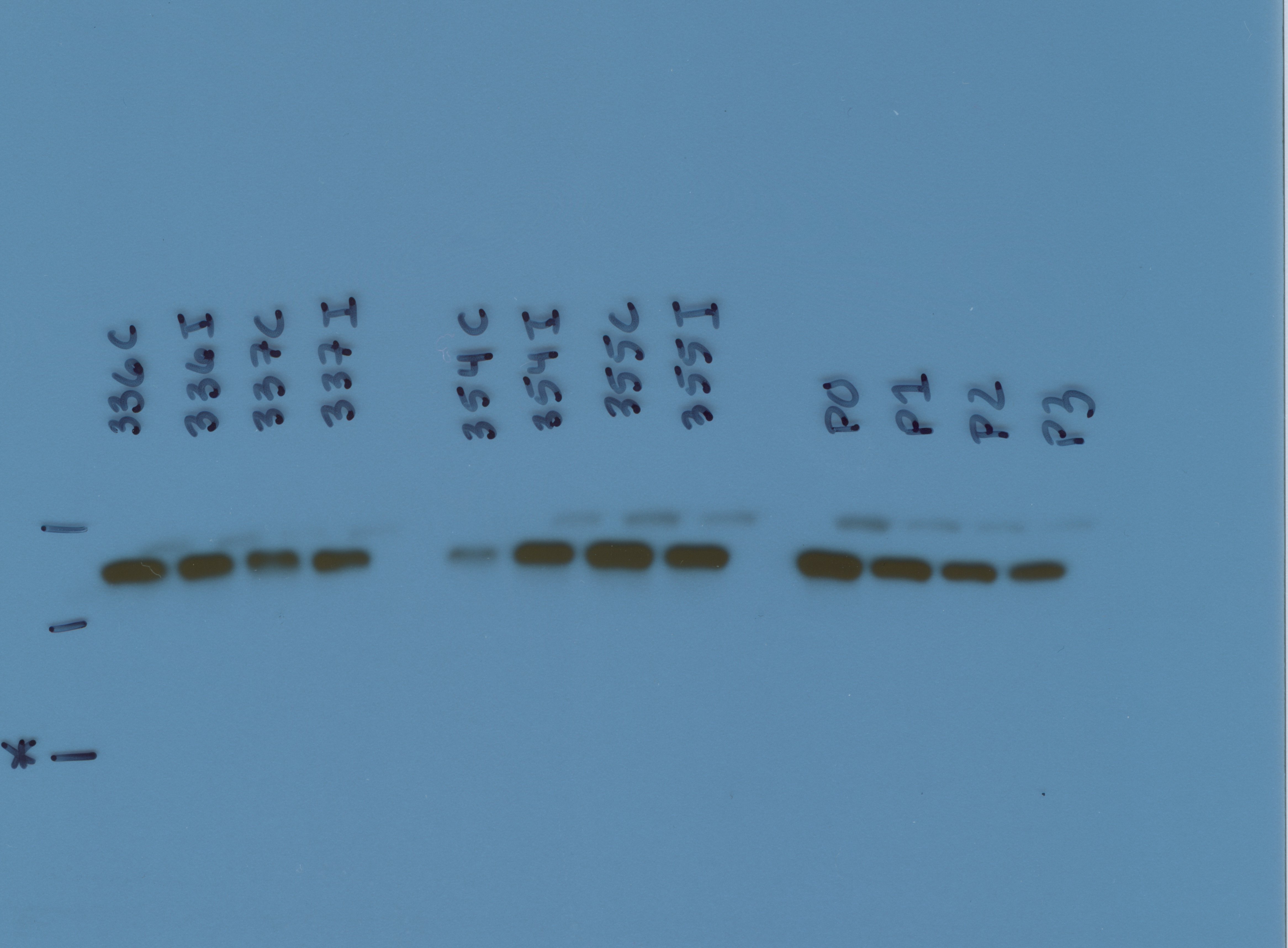

Supplement: Supplementary file 6 [file Data_Sheet_1.ZIP › 2020-8-25 336-355 P0-3 CA1 Bactin_c4.jpg]

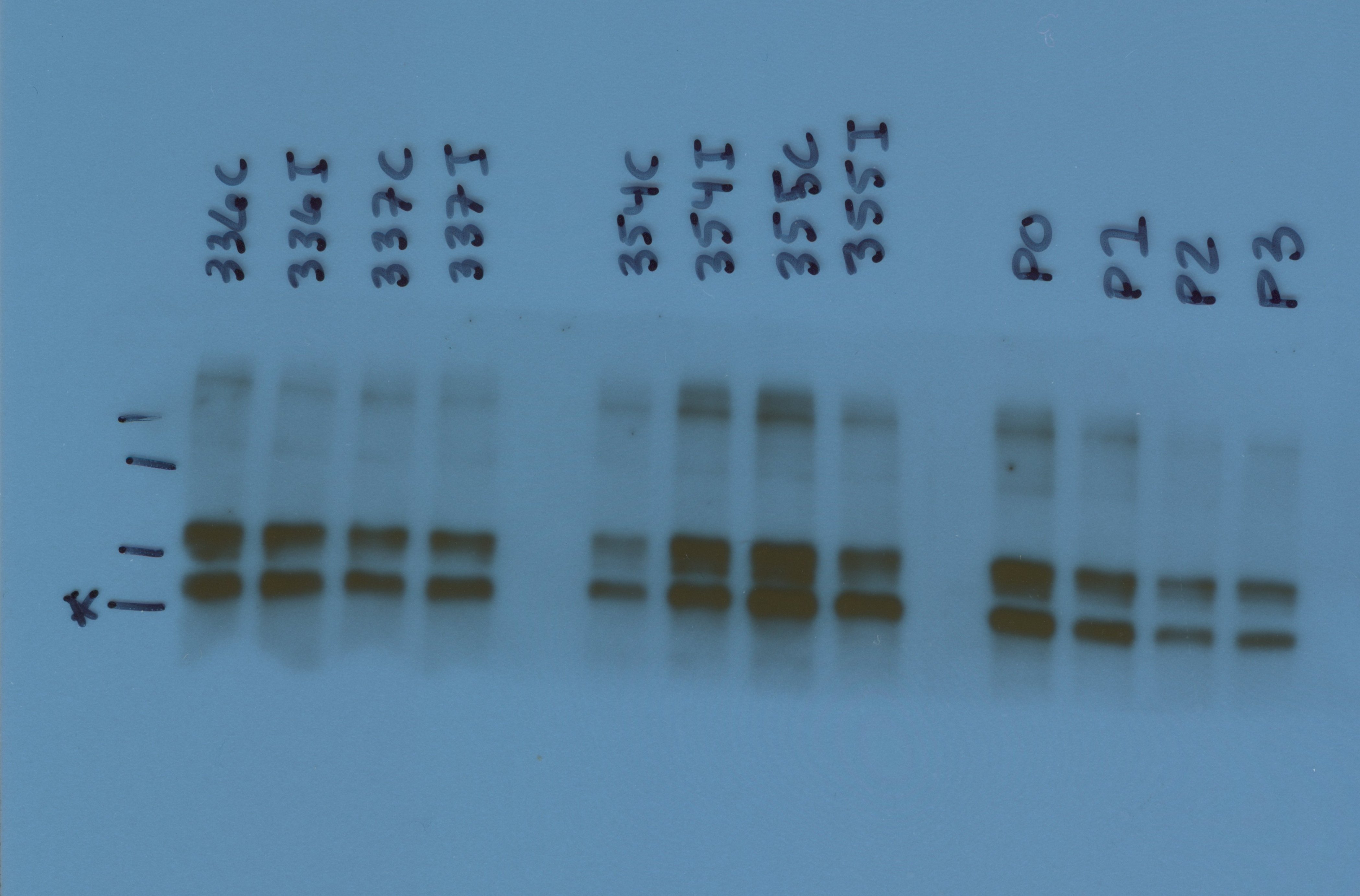

Supplement: Supplementary file 6 [file Data_Sheet_1.ZIP › 2020-8-25 336-355 P0-3 CA1 TRPC4.jpg]

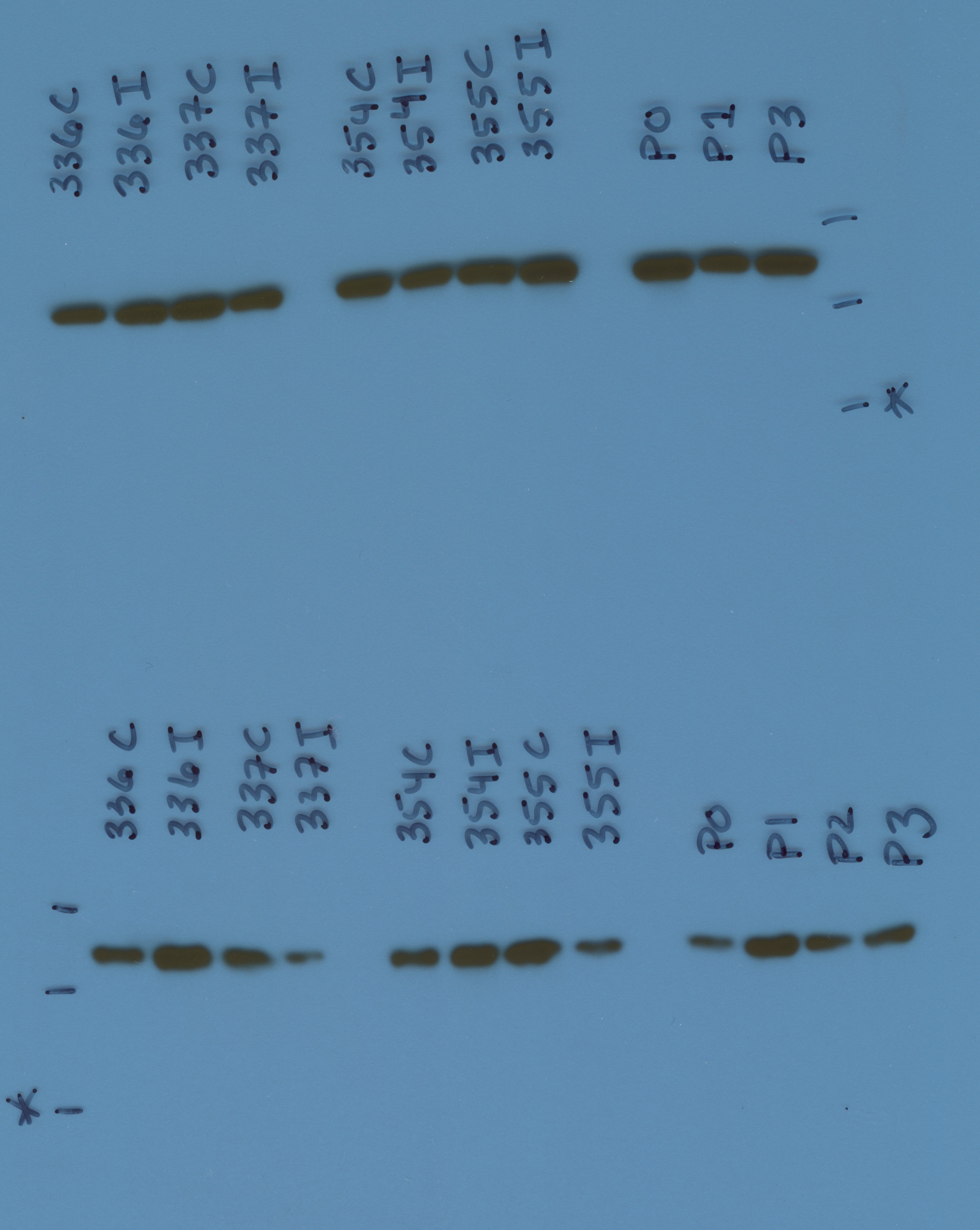

Supplement: Supplementary file 6 [file Data_Sheet_1.ZIP › 2020-8-27 336-355 P0-1 DG, CA3 BActin_c5.jpg]

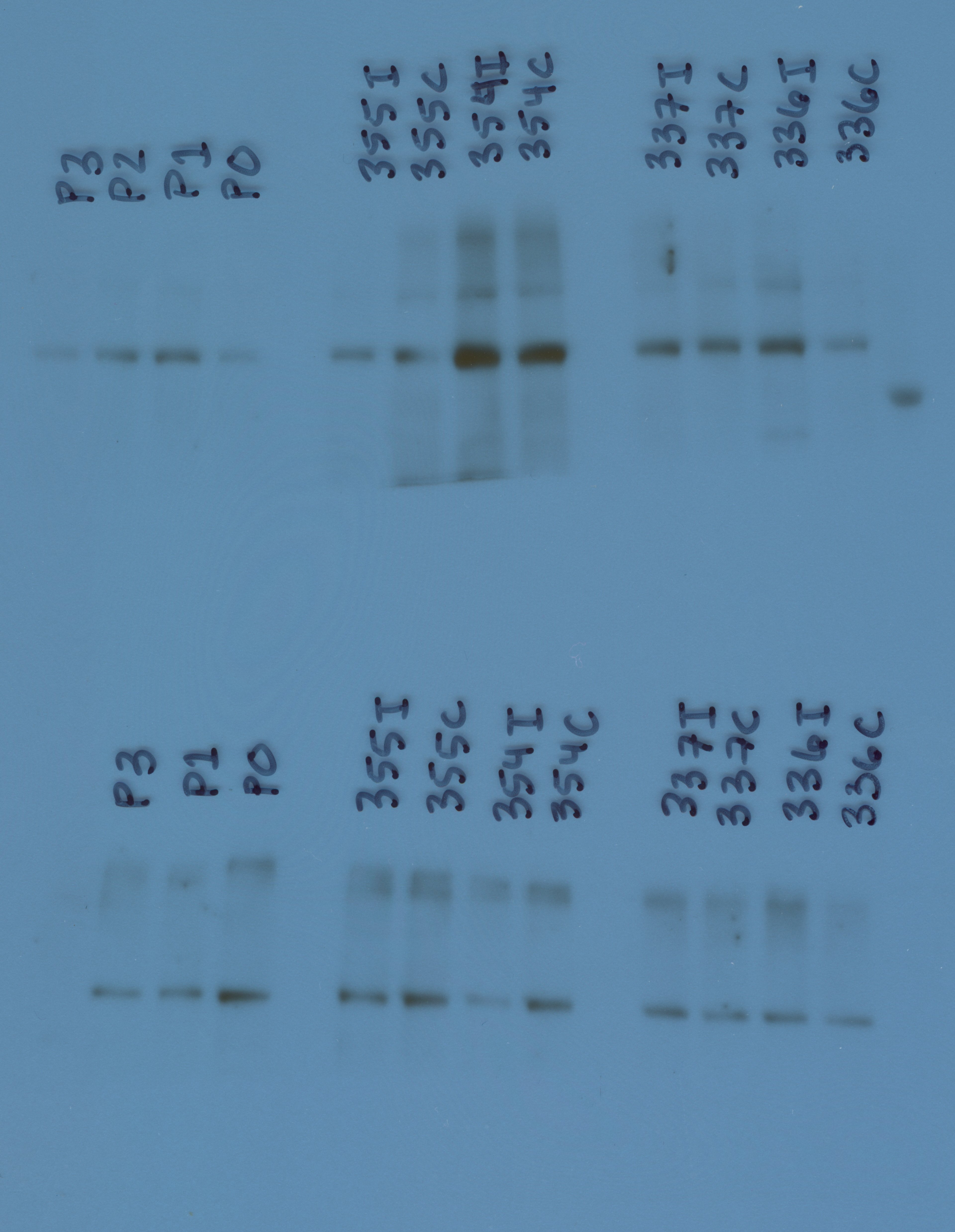

Supplement: Supplementary file 6 [file Data_Sheet_1.ZIP › 2020-8-27 336-355 P0-1 DG,CA3 TRPC5.jpg]

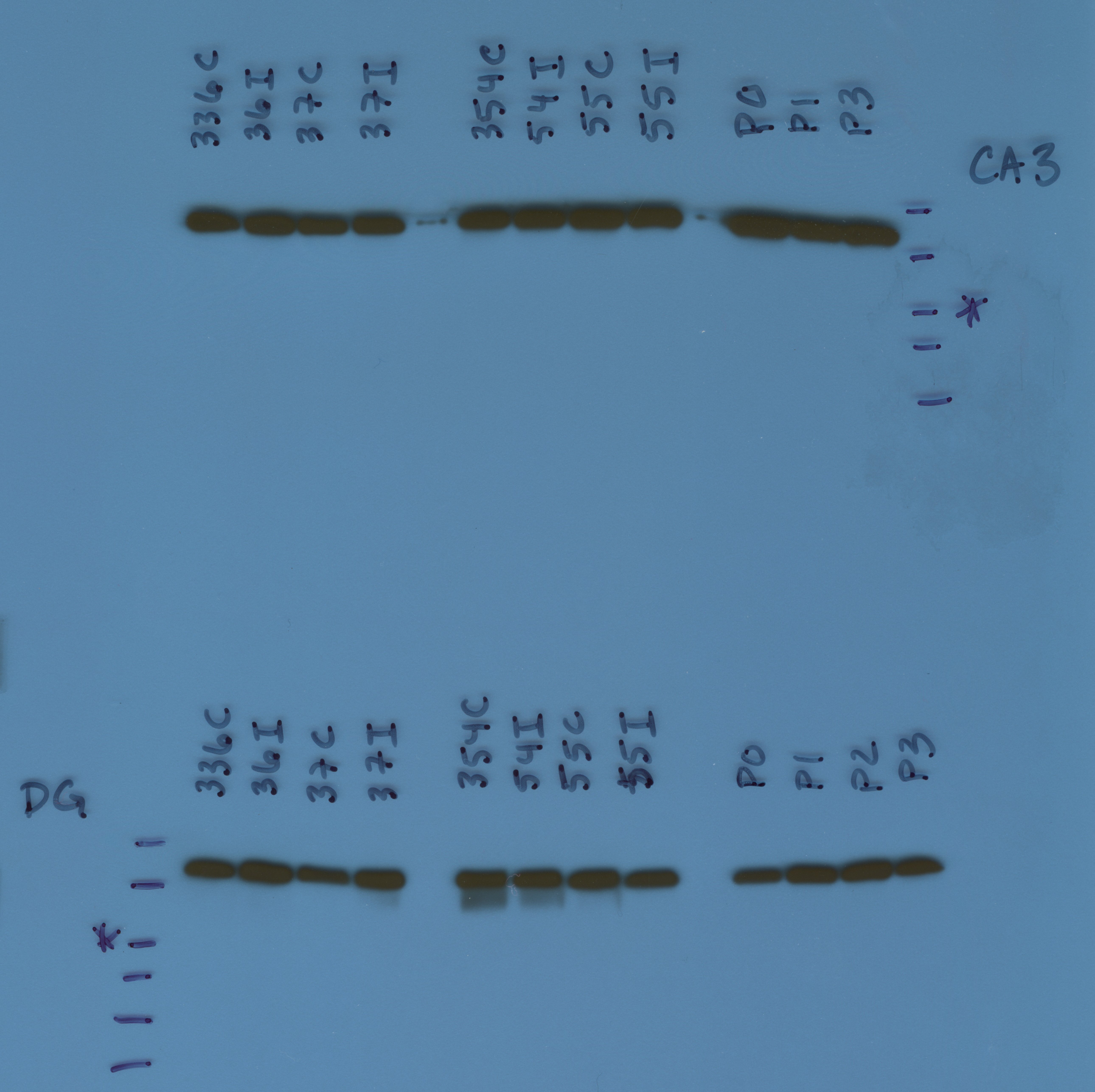

Supplement: Supplementary file 6 [file Data_Sheet_1.ZIP › 2020-9-29 336-355 P0-3 DG,CA3 Bactin_c4.jpg]

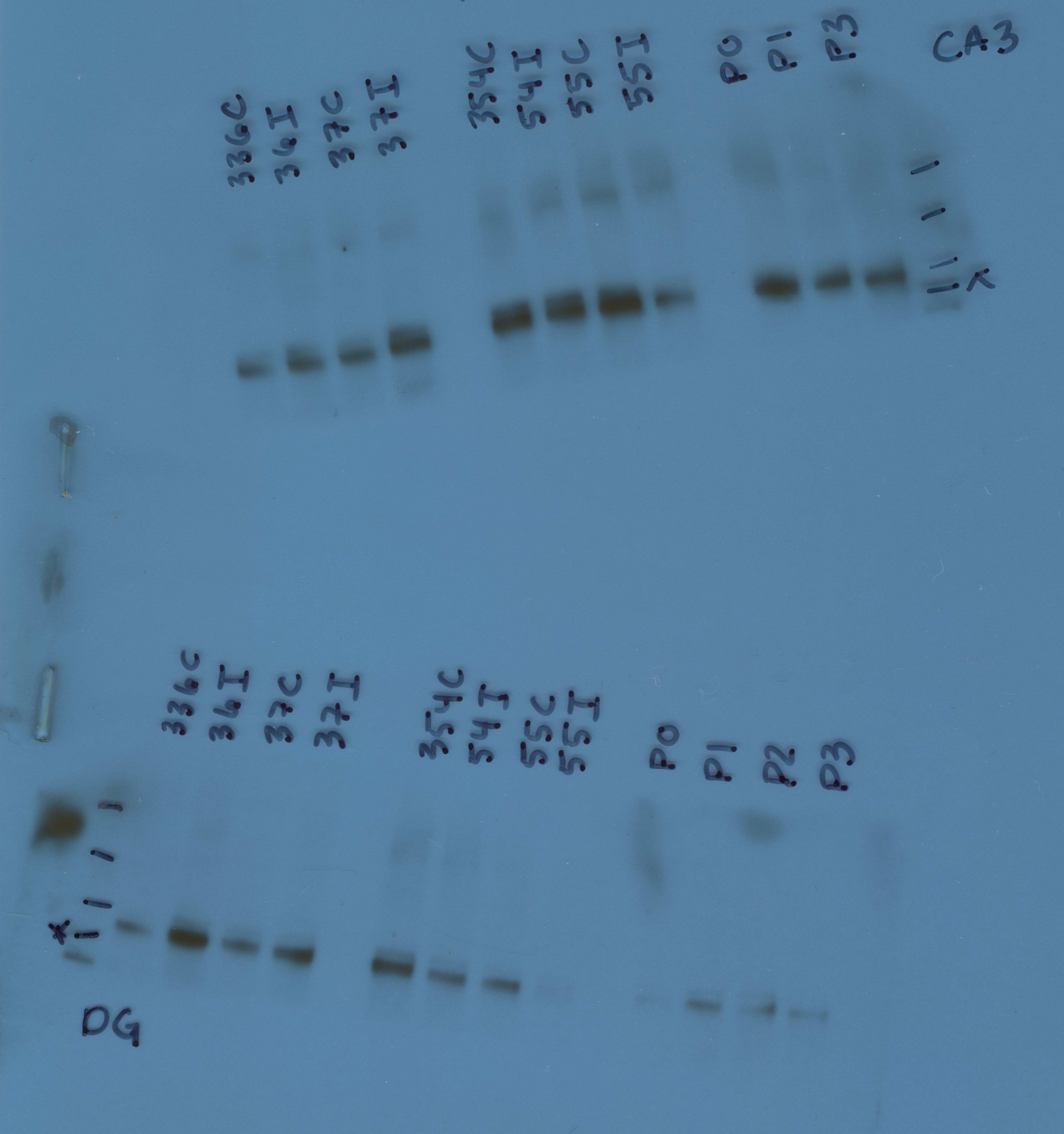

Supplement: Supplementary file 6 [file Data_Sheet_1.ZIP › 2020-9-29 336-355 P0-3 DG,CA3 TRPC4.jpg]
